# Supplementary material for: Effect of milk protein and whey permeate in large quantity lipid-based nutrient supplement on linear growth and body composition among stunted children: A randomized 2 × 2 factorial trial in Uganda
Source: PLoS Med. 2023 May 23;20(5):e1004227. doi: 10.1371/journal.pmed.1004227 (PMC10204948; doi:10.1371/journal.pmed.1004227)
Supplement: S1 Table — Effects of milk protein and whey permeate in lipid-based nutrient supplement (LNS) on linear growth and body composition among 750 children with stunting. Unadjusted analyses. (DOCX) [file pmed.1004227.s001.docx]

| **S1 Table: Intention-to-treat analysis, unadjusted. Effects of milk protein and whey permeate in lipid-based nutrient supplement (LNS) on linear growth and body composition among 750 children with stunting. Primary analysis based on the 2x2 factorial design among the 600 children given LNS and secondary analysis based on the 600 children given LNS vs 150 given no supplement. ^1^** | | | | | | | | | | |
| --- | --- | --- | --- | --- | --- | --- | --- | --- | --- | --- |
|  |  |  | **Milk vs soy protein**  **(n=299 vs n=301)** | |  | **Whey permeate vs maltodextrin**  **(n=301 vs n=299)** | |  | **LNS vs no supplement**  **(n=600 vs n=150)** | |
|  | **Interaction, p value** |  | **B (95% CI)** | **p value** |  | **B (95% CI)** | **p value** |  | **B (95% CI)** | **p value** |
| **Primary outcomes** |  |  |  |  |  |  |  |  |  |  |
| Height (cm) | 0.490 |  | 0.03 (-0.10, 0.16) | 0.659 |  | -0.08 (-0.21, 0.05) | 0.216 |  | 0.56 (0.42, 0.71) | <0.001 |
| Knee-heel length (mm) | 0.612 |  | 0.2 (-0.3, 0.7) | 0.408 |  | -0.2 (-0.7, 0.3) | 0.403 |  | 1.9 (1.4, 2.4) | <0.001 |
| **Other outcomes** |  |  |  |  |  |  |  |  |  |  |
| Height-for-age (z-score) | 0.624 |  | 0.01 (-0.03, 0.04) | 0.761 |  | -0.02 (-0.06, 0.01) | 0.182 |  | 0.17 (0.13, 0.21) | <0.001 |
| Weight (kg) | 0. 090 |  | 0.05 (-0.02, 0.12) | 0.137 |  | -0.03 (-0.09, 0.04) | 0.405 |  | 0.21 (0.14, 0.28) | <0.001 |
| Fat mass (kg) | 0.454 |  | 0.03 (-0.04, 0.09) | 0.477 |  | -0.02 (-0.09, 0.05) | 0.592 |  | 0.04 (-0.03, 0.12) | 0.253 |
| Fat-free mass (kg) | 0.290 |  | 0.04 (-0.01, 0.09) | 0.165 |  | -0.02 (-0.07, 0.03) | 0.497 |  | 0.16 (0.10, 0.21) | <0.001 |
| Fat mass index (kg/m^2^) | 0.876 |  | 0.02 (-0.09, 0.12) | 0.759 |  | -0.03 (-0.13, 0.07) | 0.558 |  | 0.02 (-0.10, 0.13) | 0.753 |
| Fat-free mass index (kg/m^2^) | 0. 633 |  | 0.05 (-0.008, 0.12) | 0.088 |  | 0.006 (-0.06, 0.07) | 0.838 |  | 0.06 (-0.01, 0.12) | 0.102 |
| Weight-for-height (z-score) | 0. 599 |  | 0.04 (-0.03, 0.11) | 0.312 |  | -0.02 (-0.09, 0.05) | 0.609 |  | 0.08 (0.004, 0.16) | 0.041 |
| Weight-for-age (z-score) | 0. 427 |  | 0.03 (-0.01, 0.08) | 0.169 |  | -0.03 (-0.08, 0.02) | 0.198 |  | 0.15 (0.10, 0.21) | <0.001 |
| Mid-upper arm circumference (cm) | 0.548 |  | 0.06 (-0.01, 0.14) | 0.108 |  | -0.03 (-0.10, 0.05) | 0.465 |  | 0.13 (0.05, 0.21) | 0.002 |
| Triceps skinfold thickness (cm) | 0.671 |  | 0.05 (-0.12, 0.23) | 0.550 |  | 0.002 (-0.17, 0.18) | 0.983 |  | 0.08 (-0.11, 0.27) | 0.410 |
| Subscapular skinfold thickness (cm) | 0.950 |  | 0.02 (-0.13, 0.18) | 0.772 |  | -0.004 (-0.16, 0.15) | 0.961 |  | -0.05 (-0.22, 0.13) | 0.604 |
| Serum insulin-like growth factor-1 (ng/ml) | 0. 593 |  | 2.19 (-0.67, 5.05) | 0.133 |  | -0.009 (-2.86, 2.84) | 0.995 |  | 3.87 (0.82, 6.93) | 0.013 |
| **^1^** Data are p value for interaction between milk protein and whey permeate, and main effect B of each intervention with 95% confidence interval and p value based on linear mixed effect models without adjustments. | | | | | | | | | | |
