# Supplementary material for: Effect of milk protein and whey permeate in large quantity lipid-based nutrient supplement on linear growth and body composition among stunted children: A randomized 2 × 2 factorial trial in Uganda
Source: PLoS Med. 2023 May 23;20(5):e1004227. doi: 10.1371/journal.pmed.1004227 (PMC10204948; doi:10.1371/journal.pmed.1004227)
Supplement: S2 Table — Adjusted and unadjusted analyses. (DOCX) [file pmed.1004227.s002.docx]

| S2 Table: Adjusted analysis: Subgroup effects of milk protein in lipid-based nutrient supplement on growth by sex, breastfeeding status, stunting severity and inflammation among children with stunting who received lipid-based nutrient supplement (n=600). Data shown are p for interaction and stratum-specific effect estimates (95% confidence interval) ^1^ | | | | | | | | | | | | |
| --- | --- | --- | --- | --- | --- | --- | --- | --- | --- | --- | --- | --- |
| Outcome | **Sex** | |  | **Breastfeeding** | |  | **Stunting severity** | | |  | **Inflammation** | |
|  | Boy (n=328) | |  | Not breastfeeding (n=518) | |  | Moderate (n=342) | | |  | No inflammation (n=210) | |
|  | Girl (n=272) | |  | Breastfeeding (n=80) | |  | Severe (n=258) | | |  | Inflammation (n=381) | |
|  | **Interaction, p** | **B (95% CI)** |  | **Interaction, p** | **B (95% CI)** |  | | **Interaction, p** | **B (95% CI)** |  | **Interaction, p** | **B (95% CI)** |
| Height (cm) | 0.107 | 0.13 (-0.05, 0.30) |  | 0.678 | 0.02 (-0.12, 0.16) |  | | 0.209 | 0.10 (-0.07, 0.27) |  | 0.024 | -0.10 (-0.29, 0.08) |
|  |  | -0.09 (-0.28, 0.10) |  |  | 0.10 (-0.26, 0.46) |  | |  | -0.07 (-0.26, 0.13) |  |  | 0.20 (0.01, 0.38) |
| Knee-heel length (mm) | 0.071 | 0.6 (-0.02, 1.2) |  | 0.237 | 0.1 (-0.4, 0.6) |  | | 0.689 | 0.3 (-0.3, 0.9) |  | 0.440 | 0.1 (-0.6, 0.7) |
|  |  | -0.3 (-1.0, 0.4) |  |  | 0.9 (-0.4, 2.2) |  | |  | 0.1 (-0.6, 0.8) |  |  | 0.4 (-0.2, 1.1) |
| Height-for-age (z-score) | 0.114 | 0.04 (-0.02, 0.09) |  | 0.676 | 0.01 (-0.03, 0.04) |  | | 0.260 | 0.03 (-0.02, 0.08) |  | 0.057 | -0.03 (-0.08, 0.03) |
|  |  | -0.03 (-0.08, 0.03) |  |  | 0.03 (-0.07, 0.13) |  | |  | -0.02 (-0.07, 0.04) |  |  | 0.05 (-0.006, 0.10) |
| Weight (kg) | 0.766 | 0.05 (-0.04, 0.14) |  | 0.668 | 0.07 (0.002, 0.14) |  | | 0.035 | 0.12 (0.03, 0.20) |  | 0.546 | 0.03 (-0.06, 0.12) |
|  |  | 0.07 (-0.03, 0.16) |  |  | 0.03 (-0.15, 0.21) |  | |  | -0.02 (-0.12, 0.08) |  |  | 0.07 (-0.02, 0.16) |
| Fat mass (kg) | 0.229 | -0.01 (-0.10, 0.09) |  | 0.317 | 0.06 (-0.01, 0.13) |  | | 0.355 | 0.06 (-0.03, 0.15) |  | 0.878 | 0.03 (-0.08, 0.14) |
|  |  | 0.08 (-0.02, 0.18) |  |  | -0.04 (-0.22, 0.15) |  | |  | -0.004 (-0.11, 0.10) |  |  | 0.02 (-0.06, 0.10) |
| Fat-free mass (kg) | 0.231 | 0.06 (-0.004, 0.13) |  | 0.483 | 0.03 (-0.03, 0.08) |  | | 0.171 | 0.06 (-0.001, 0.13) |  | 0.196 | -0.01 (-0.09, 0.07) |
|  |  | 0.002 (-0.07, 0.07) |  |  | 0.07 (-0.06, 0.21) |  | |  | -0.005 (-0.08, 0.07) |  |  | 0.06 (-0.001, 0.12) |
| Fat mass index (kg/m^2^) | 0.205 | -0.03 (-0.17, 0.11) |  | 0.476 | 0.07 (-0.04, 0.18) |  | | 0.348 | 0.07 (-0.06, 0.21) |  | 0.599 | 0.05 (-0.12, 0.22) |
|  |  | 0.10 (-0.05, 0.25) |  |  | -0.04 (-0.31, 0.24) |  | |  | -0.02 (-0.18, 0.13) |  |  | -0.004 (-0.13, 0.12) |
| Fat-free mass index (kg/m^2^) | 0.948 | 0.06 (-0.02, 0.14) |  | 0.322 | 0.04 (-0.02, 0.11) |  | | 0.999 | 0.06 (-0.02, 0.14) |  | 0.504 | 0.02 (-0.08, 0.13) |
|  |  | 0.06 (-0.04, 0.15) |  |  | 0.13 (-0.04, 0.30) |  | |  | 0.06 (-0.03, 0.15) |  |  | 0.07 (-0.01, 0.14) |
| Weight-for-height (z-score) | 0.328 | 0.01 (-0.08, 0.11) |  | 0.963 | 0.05 (-0.03, 0.12) |  | | 0.160 | 0.09 (-0.01, 0.18) |  | 0.983 | 0.03 (-0.07, 0.13) |
|  |  | 0.08 (-0.02, 0.19) |  |  | 0.04 (-0.15, 0.24) |  | |  | -0.02 (-0.12, 0.09) |  |  | 0.03 (-0.06, 0.13) |
| Weight-for-age (z-score) | 0.884 | 0.04 (-0.03, 0.10) |  | 0.957 | 0.05 (-0.01, 0.10) |  | | 0.091 | 0.08 (0.01, 0.14) |  | 0.299 | 0.01 (-0.06, 0.08) |
|  |  | 0.05 (-0.03, 0.12) |  |  | 0.04 (-0.09, 0.18) |  | |  | -0.01 (-0.08, 0.07) |  |  | 0.06 (-0.01, 0.13) |
| Mid-upper arm circumference (cm) | 0.596 | 0.08 (-0.02, 0.18) |  | 0.692 | 0.07 (-0.01, 0.15) |  | | 0.019 | 0.14 (0.04, 0.24) |  | 0.365 | 0.02 (-0.08, 0.12) |
|  |  | 0.04 (-0.07, 0.15) |  |  | 0.03 (-0.18, 0.23) |  | |  | -0.04 (-0.15, 0.07) |  |  | 0.09 (-0.02, 0.19) |
| Triceps skinfold (mm) | 0.657 | 0.03 (-0.19, 0.25) |  | 0.675 | 0.09 (-0.09, 0.27) |  | | 0.216 | 0.16 (-0.06, 0.37) |  | 0.806 | 0.08 (-0.16, 0.31) |
|  |  | 0.10 (-0.14, 0.35) |  |  | -0.01 (-0.47, 0.44) |  | |  | -0.05 (-0.30, 0.20) |  |  | 0.03 (-0.20, 0.27) |
| Subscapular skinfold (mm) | 0.449 | 0.08 (-0.13, 0.29) |  | 0.791 | 0.03 (-0.14, 0.19) |  | | 0.096 | 0.14 (-0.06, 0.35) |  | 0.506 | -0.03 (-0.25, 0.18) |
|  |  | -0.04 (-0.27, 0.19) |  |  | 0.09 (-0.34, 0.52) |  | |  | -0.12 (-0.36, 0.11) |  |  | 0.07 (-0.15, 0.29) |
| Insulin-like growth factor-1 (ng/ml) | 0.834 | 1.97 (-1.75, 5.68) |  | 0.385 | 2.60 (-0.35, 5.54) |  | | 0.473 | 3.10 (-0.51, 6.71) |  | 0.739 | 2.72 (-1.17, 6.61) |
|  |  | 2.55 (-1.50, 6.60) |  |  | -1.03 (-8.67, 6.60) |  | |  | 1.08 (-3.09, 5.24) |  |  | 1.78 (-2.12, 5.68) |
| ^1^ Based on linear mixed effect models adjusted for age, sex, season and site. | | | | | | | | | | | | |

| S2 Table: Unadjusted analysis: Subgroup effects of milk protein in lipid-based nutrient supplement on growth by sex, breastfeeding status, stunting severity and inflammation among children with stunting who received lipid-based nutrient supplement (n=600). Data shown are p for interaction and stratum-specific effect estimates (95% confidence interval) ^1^ | | | | | | | | | | | | |
| --- | --- | --- | --- | --- | --- | --- | --- | --- | --- | --- | --- | --- |
| Outcome | **Sex** | |  | **Breastfeeding** | |  | **Stunting severity** | | |  | **Inflammation** | |
|  | Boy (n=328) | |  | Not breastfeeding (n=518) | |  | Moderate (n=342) | | |  | No inflammation (n=210) | |
|  | Girl (n=272) | |  | Breastfeeding (n=80) | |  | Severe (n=258) | | |  | Inflammation (n=381) | |
|  | **Interaction, p** | **B (95% CI)** |  | **Interaction, p** | **B (95% CI)** |  | | **Interaction, p** | **B (95% CI)** |  | **Interaction, p** | **B (95% CI)** |
| Height (cm) | 0.115 | 0.13 (-0.05, 0.30) |  | 0.757 | 0.02 (-0.12, 0.16) |  | | 0.315 | 0.09 (-0.08, 0.26) |  | 0.021 | -0.11 (-0.29, 0.08) |
|  |  | -0.08 (-0.28, 0.11) |  |  | 0.10 (-0.28, 0.45) |  | |  | -0.05 (-0.25, 0.15) |  |  | 0.20 (0.02, 0.39) |
| Knee-heel length (mm) | 0.053 | 0.7 (0.01, 1.3) |  | 0.208 | 0.1 (-0.4, 0.6) |  | | 0.673 | 0.3 (-0.3, 0.9) |  | 0.372 | 0.03 (-0.7, 0.7) |
|  |  | -0.3 (-1.0, 0.4) |  |  | 1.0 (-0.3, 2.3) |  | |  | 0.1 (-0.6, 0.8) |  |  | 0.5 (-0.2, 1.1) |
| Height-for-age (z-score) | 0.117 | 0.04 (-0.02, 0.09) |  | 0.760 | 0.005 (-0.04, 0.05) |  | | 0.276 | 0.03 (-0.02, 0.08) |  | 0.055 | -0.03 (-0.08, 0.03) |
|  |  | -0.03 (-0.08, 0.03) |  |  | 0.02 (-0.08, 0.13) |  | |  | -0.02 (-0.07, 0.04) |  |  | 0.05 (-0.005, 0.10) |
| Weight (kg) | 0.765 | 0.04 (-0.04, 0.13) |  | 0.810 | 0.06 (-0.01, 0.13) |  | | 0.077 | 0.10 (0.02, 0.19) |  | 0.484 | 0.02 (-0.07, 0.11) |
|  |  | 0.07 (-0.03, 0.16) |  |  | 0.04 (-0.14, 0.22) |  | |  | -0.02 (-0.12, 0.08) |  |  | 0.07 (-0.02, 0.16) |
| Fat mass (kg) | 0.216 | -0.01 (-0.11, 0.08) |  | 0.513 | 0.04 (-0.03, 0.12) |  | | 0.209 | 0.06 (-0.03, 0.16) |  | 0.900 | 0.03 (-0.07, 0.12) |
|  |  | 0.07 (-0.03, 0.18) |  |  | -0.02 (-0.21, 0.16) |  | |  | -0.03 (-0.13, 0.08) |  |  | 0.02 (-0.08, 0.11) |
| Fat-free mass (kg) | 0.199 | 0.07 (-0.002, 0.14) |  | 0.432 | 0.03 (-0.03, 0.08) |  | | 0.313 | 0.06 (-0.01, 0.13) |  | 0.131 | 0.001 (-0.07, 0.07) |
|  |  | 0.001 (-0.08, 0.08) |  |  | 0.09 (-0.05, 0.23) |  | |  | 0.01 (-0.07, 0.08) |  |  | 0.08 (0.01, 0.15) |
| Fat mass index (kg/m^2^) | 0.276 | -0.03 (-0.17, 0.11) |  | 0.714 | 0.03 (-0.08, 0.14) |  | | 0.295 | 0.07 (-0.07, 0.20) |  | 0.819 | 0.02 (-0.12, 0.17) |
|  |  | 0.08 (-0.07, 0.24) |  |  | -0.03 (-0.31, 0.26) |  | |  | -0.05 (-0.20, 0.11) |  |  | -0.001 (-0.14, 0.14) |
| Fat-free mass index (kg/m^2^) | 0.866 | 0.06 (-0.02, 0.14) |  | 0.274 | 0.04 (-0.03, 0.10) |  | | 0.954 | 0.05 (-0.03, 0.14) |  | 0.926 | 0.05 (-0.04, 0.14) |
|  |  | 0.07 (-0.02, 0.16) |  |  | 0.14 (-0.03, 0.31) |  | |  | 0.06 (-0.04, 0.15) |  |  | 0.06 (-0.03, 0.14) |
| Weight-for-height (z-score) | 0.275 | 0.01 (-0.09, 0.10) |  | 0.829 | 0.04 (-0.04, 0.12) |  | | 0.181 | 0.08 (-0.01, 0.18) |  | 0.931 | 0.03 (-0.07, 0.13) |
|  |  | 0.09 (-0.02, 0.19) |  |  | 0.06 (-0.13, 0.26) |  | |  | -0.02 (-0.12, 0.09) |  |  | 0.04 (-0.06, 0.13) |
| Weight-for-age (z-score) | 0.794 | 0.03 (-0.03, 0.10) |  | 0.828 | 0.04 (-0.02, 0.09) |  | | 0.087 | 0.08 (0.01, 0.14) |  | 0.288 | 0.01 (-0.06, 0.08) |
|  |  | 0.05 (-0.03, 0.12) |  |  | 0.05 (-0.08, 0.19) |  | |  | -0.01 (-0.09, 0.06) |  |  | 0.06 (-0.01, 0.13) |
| Mid-upper arm circumference (cm) | 0.590 | 0.08 (-0.02, 0.18) |  | 0.698 | 0.07 (-0.01, 0.15) |  | | 0.036 | 0.13 (0.03, 0.23) |  | 0.311 | 0.02 (-0.09, 0.12) |
|  |  | 0.04 (-0.07, 0.15) |  |  | 0.03 (-0.18, 0.23) |  | |  | -0.03 (-0.14, 0.09) |  |  | 0.09 (-0.01, 0.20) |
| Triceps skinfold (mm) | 0.595 | 0.01 (-0.23, 0.24) |  | 0.677 | 0.07 (-0.12, 0.26) |  | | 0.127 | 0.18 (-0.05, 0.40) |  | 0.967 | 0.05 (-0.19, 0.29) |
|  |  | 0.10 (-0.15, 0.36) |  |  | -0.04 (-0.52, 0.44) |  | |  | -0.10 (-0.36, 0.17) |  |  | 0.04 (-0.20, 0.28) |
| Subscapular skinfold (mm) | 0.546 | 0.06 (-0.15, 0.27) |  | 0.647 | 0.01 (-0.16, 0.18) |  | | 0.109 | 0.14 (-0.07, 0.34) |  | 0.492 | -0.04 (-0.25, 0.18) |
|  |  | -0.03 (-0.26, 0.20) |  |  | 0.12 (-0.31, 0.55) |  | |  | -0.12 (-0.36, 0.12) |  |  | 0.07 (-0.14, 0.29) |
| Insulin-like growth factor-1 (ng/ml) | 0.868 | 1.91 (-1.94, 5.77) |  | 0.386 | 2.85 (-0.21, 5.91) |  | | 0.841 | 2.43 (-1.34, 6.21) |  | 0.920 | 2.24 (-1.81, 6.29) |
|  |  | 2.39 (-1.83, 6.61) |  |  | -0.90 (-8.81, 7.02) |  | |  | 1.85 (-2.49, 6.19) |  |  | 1.94 (-2.12, 6.01) |
| ^1^ Based on linear mixed effect models without adjustments. | | | | | | | | | | | | |
