# Supplementary material for: Effect of milk protein and whey permeate in large quantity lipid-based nutrient supplement on linear growth and body composition among stunted children: A randomized 2 × 2 factorial trial in Uganda
Source: PLoS Med. 2023 May 23;20(5):e1004227. doi: 10.1371/journal.pmed.1004227 (PMC10204948; doi:10.1371/journal.pmed.1004227)
Supplement: S3 Table — Adjusted and unadjusted analyses. (DOCX) [file pmed.1004227.s003.docx]

| S3 Table: Adjusted analysis: Subgroup effects of whey permeate in lipid-based nutrient supplement on growth by sex, breastfeeding status, stunting severity and inflammation among children with stunting who received lipid-based nutrient supplement (n=600). Data shown are p for interaction and stratum-specific effect estimates (95% confidence interval) ^1^ | | | | | | | | | | | | |
| --- | --- | --- | --- | --- | --- | --- | --- | --- | --- | --- | --- | --- |
| Outcome | **Sex** | |  | **Breastfeeding** | |  | **Stunting severity** | | |  | **Inflammation** | |
|  | Boy (n=328) | |  | Not breastfeeding (n=518) | |  | Moderate (n=342) | | |  | No inflammation (n=210) | |
|  | Girl (n=272) | |  | Breastfeeding (n=80) | |  | Severe (n=258) | | |  | Inflammation (n=381) | |
|  | **Interaction, p** | **B (95% CI)** |  | **Interaction, p** | **B (95% CI)** |  | | **Interaction, p** | **B (95% CI)** |  | **Interaction, p** | **B (95% CI)** |
| Height (cm) | 0.460 | -0.13 (-0.30, 0.05) |  | 0.003 | -0.16 (-0.30, -0.02) |  | | 0.818 | -0.07 (-0.24, 0.10) |  | 0.780 | -0.06 (-0.24, 0.13) |
|  |  | -0.03 (-0.22, 0.16) |  |  | 0.44 (0.08, 0.81) |  | |  | -0.10 (-0.29, 0.10) |  |  | -0.09 (-0.28, 0.09) |
| Knee-heel length (mm) | 0.345 | 0.01 (-0.6, 0.6) |  | 0.016 | -0.4 (-0.9, 0.1) |  | | 0.445 | -0.3 (-0.9, 0.3) |  | 0.053 | 0.3 (-0.4, 1.0) |
|  |  | -0.4 (-1.1, 0.2) |  |  | 1.3 (0.01, 2.7) |  | |  | 0.1 (-0.7, 0.8) |  |  | -0.6 (-1.3, 0.02) |
| Height-for-age (z-score) | 0.511 | -0.03 (-0.09, 0.02) |  | 0.003 | -0.04 (-0.08, 0.002) |  | | 0.815 | -0.02 (-0.07, 0.03) |  | 0.696 | -0.03 (-0.08, 0.03) |
|  |  | -0.01 (-0.07, 0.05) |  |  | 0.13 (0.03, 0.24) |  | |  | -0.03 (-0.09, 0.03) |  |  | -0.01 (-0.07, 0.04) |
| Weight (kg) | 0.058 | 0.02 (-0.06, 0.11) |  | 0.677 | -0.03 (-0.09, 0.04) |  | | 0.698 | -0.05 (-0.13, 0.04) |  | 0.256 | 0.01 (-0.08, 0.10) |
|  |  | -0.10 (-0.20, -0.005) |  |  | 0.01 (-0.17, 0.20) |  | |  | -0.02 (-0.12, 0.08) |  |  | -0.07 (-0.16, 0.02) |
| Fat mass (kg) | 0.053 | 0.05 (-0.05, 0.14) |  | 0.748 | 0.004 (-0.07, 0.08) |  | | 0.372 | 0.01 (-0.08, 0.10) |  | 0.993 | -0.02 (-0.13, 0.09) |
|  |  | -0.09 (-0.19, 0.01) |  |  | 0.04 (-0.15, 0.23) |  | |  | -0.05 (-0.16, 0.05) |  |  | -0.02 (-0.10, 0.07) |
| Fat-free mass (kg) | 0.618 | - 0.03 (-0.10, 0.03) |  | 0.894 | -0.02 (-0.07, 0.03) |  | | 0.194 | -0.05 (-0.11, 0.02) |  | 0.703 | -0.01 (-0.09, 0.07) |
|  |  | -0.01 (-0.08, 0.06) |  |  | -0.03 (-0.17, 0.11) |  | |  | 0.02 (-0.06, 0.10) |  |  | -0.03 (-0.09, 0.03) |
| Fat mass index (kg/m^2^) | 0.051 | 0.07 (-0.06, 0.21) |  | 0.882 | 0.02 (-0.09, 0.12) |  | | 0.381 | 0.02 (-0.12, 0.15) |  | 0.894 | -0.03 (-0.20, 0.13) |
|  |  | -0.13 (-0.28, 0.02) |  |  | 0.04 (-0.24, 0.32) |  | |  | -0.08 (-0.23, 0.08) |  |  | -0.02 (-0.14, 0.10) |
| Fat-free mass index (kg/m^2^) | 0.519 | 0.04 (-0.05, 0.12) |  | 0.018 | 0.04 (-0.02, 0.11) |  | | 0.028 | -0.05 (-0.13, 0.03) |  | 0.717 | 0.03 (-0.07, 0.13) |
|  |  | -0.004 (-0.09, 0.09) |  |  | -0.18 (-0.35, -0.01) |  | |  | 0.09 (-0.00, 0.18) |  |  | 0.01 (-0.07, 0.08) |
| Weight-for-height (z-score) | 0.008 | 0.07 (-0.03, 0.16) |  | 0.522 | 0.004 (-0.07, 0.08) |  | | 0.925 | -0.03 (-0.12, 0.06) |  | 0.417 | 0.01 (-0.09, 0.11) |
|  |  | -0.12 (-0.23, -0.02) |  |  | -0.07 (-0.27, 0.13) |  | |  | -0.02 (-0.13, 0.09) |  |  | -0.05 (-0.14, 0.05) |
| Weight-for-age (z-score) | 0.031 | 0.02 (-0.05, 0.09) |  | 0.558 | -0.02 (-0.08, 0.03) |  | | 0.890 | -0.03 (-0.10, 0.04) |  | 0.376 | -0.01 (-0.08, 0.06) |
|  |  | -0.09 (-0.16, -0.02) |  |  | 0.02 (-0.12, 0.16) |  | |  | -0.04 (-0.11, 0.04) |  |  | -0.05 (-0.12, 0.02) |
| Mid-upper arm circumference (cm) | 0.991 | -0.03 (-0.13, 0.07) |  | 0.515 | -0.02 (-0.10, 0.06) |  | | 0.200 | -0.07 (-0.17, 0.03) |  | 0.463 | -0.06 (-0.16, 0.04) |
|  |  | -0.03 (-0.14, 0.08) |  |  | 0.05 (-0.16, 0.26) |  | |  | 0.03 (-0.09, 0.14) |  |  | -0.004 (-0.11, 0.10) |
| Triceps skinfold (mm) | 0.462 | 0.05 (-0.17, 0.28) |  | 0.874 | 0.03 (-0.14, 0.21) |  | | 0.842 | -0.02 (-0.24, 0.19) |  | 0.826 | 0.02 (-0.21, 0.25) |
|  |  | -0.07 (-0.31, 0.17) |  |  | -0.01 (-0.47, 0.46) |  | |  | 0.01 (-0.24, 0.26) |  |  | -0.02 (-0.25, 0.21) |
| Subscapular skinfold (mm) | 0.126 | 0.11 (-0.10, 0.32) |  | 0.774 | 0.02 (-0.15, 0.19) |  | | 0.503 | -0.06 (-0.26, 0.15) |  | 0.471 | -0.06 (-0.27, 0.16) |
|  |  | -0.13 (-0.36, 0.10) |  |  | -0.05 (-0.49, 0.39) |  | |  | 0.05 (-0.19, 0.29) |  |  | 0.06 (-0.16, 0.27) |
| Insulin-like growth factor-1 (ng/ml) | 0.965 | -0.86 (-4.57, 2.86) |  | 0.529 | -1.29 (-4.26, 1.68) |  | | 0.044 | 1.67 (-1.95, 5.29) |  | 0.974 | -0.92 (-4.82, 2.98) |
|  |  | -0.98 (-5.05, 3.10) |  |  | 1.41 (-6.44, 9.27) |  | |  | -4.04 (-8.24, 0.16) |  |  | -0.83 (-4.73, 3.08) |
| ^1^ Based on linear mixed effect models adjusted for age, sex, season and site. | | | | | | | | | | | | |

| S3 Table: Unadjusted analysis: Subgroup effects of whey permeate in lipid-based nutrient supplement on growth by sex, breastfeeding status, stunting severity and inflammation among children with stunting who received lipid-based nutrient supplement (n=600). Data shown are p for interaction and stratum-specific effect estimates (95% confidence interval) ^1^ | | | | | | | | | | | | |
| --- | --- | --- | --- | --- | --- | --- | --- | --- | --- | --- | --- | --- |
| Outcome | **Sex** | |  | **Breastfeeding** | |  | **Stunting severity** | | |  | **Inflammation** | |
|  | Boy (n=328) | |  | Not breastfeeding (n=518) | |  | Moderate (n=342) | | |  | No inflammation (n=210) | |
|  | Girl (n=272) | |  | Breastfeeding (n=80) | |  | Severe (n=258) | | |  | Inflammation (n=381) | |
|  | **Interaction, p** | **B (95% CI)** |  | **Interaction, p** | **B (95% CI)** |  | | **Interaction, p** | **B (95% CI)** |  | **Interaction, p** | **B (95% CI)** |
| Height (cm) | 0.602 | -0.11 (-0.29, 0.06) |  | 0.002 | -0.16 (-0.30, -0.02) |  | | 0.683 | -0.06 (-0.23, 0.11) |  | 0.683 | -0.06 (-0.23, 0.11) |
|  |  | -0.04 (-0.24, 0.15) |  |  | 0.46 (0.09, 0.82) |  | |  | -0.11 (-0.31, 0.09) |  |  | -0.11 (-0.31, 0.09) |
| Knee-heel length (mm) | 0.537 | 0.05 (-0.7, 0.6) |  | 0.074 | -0.4 (-0.9, 0.2) |  | | 0.708 | -0.3 (-0.9, 0.4) |  | 0.043 | 0.3 (-0.4, 1.0) |
|  |  | -0.4 (-1.1, 0.4) |  |  | 1.0 (-0.4, 2.3) |  | |  | 0.1 (-0.8, 0.6) |  |  | -0.7 (-1.3, 0.01) |
| Height-for-age (z-score) | 0.661 | -0.03 (-0.08, 0.02) |  | 0.002 | -0.05 (-0.09, -0.004) |  | | 0.663 | -0.02 (-0.07, 0.03) |  | 0.652 | -0.03 (-0.09, 0.02) |
|  |  | -0.02 (-0.07, 0.04) |  |  | 0.14 (0.03, 0.25) |  | |  | -0.04 (-0.09, 0.02) |  |  | -0.01 (-0.07, 0.04) |
| Weight (kg) | 0.067 | 0.03 (-0.06, 0.11) |  | 0.743 | -0.02 (-0.09, 0.05) |  | | 0.708 | -0.04 (-0.13, 0.04) |  | 0.295 | 0.01 (-0.08, 0.10) |
|  |  | -0.09 (-0.19, 0.004) |  |  | 0.02 (-0.17, 0.20) |  | |  | -0.02 (-0.12, 0.08) |  |  | -0.06 (-0.15, 0.03) |
| Fat mass (kg) | 0.095 | 0.04 (-0.06, 0.13) |  | 0.803 | -0.01 (-0.08, 0.06) |  | | 0.555 | -0.003 (-0.09, 0.09) |  | 0.501 | 0.01 (-0.09, 0.10) |
|  |  | -0.08 (-0.18, 0.02) |  |  | 0.02 (-0.18, 0.21) |  | |  | -0.05 (-0.15, 0.06) |  |  | -0.04 (-0.13, 0.05) |
| Fat-free mass (kg) | 0.826 | - 0.02 (-0.09, 0.05) |  | 0.923 | -0.02 (-0.07, 0.04) |  | | 0.205 | -0.05 (-0.12, 0.02) |  | 0.641 | -0.004 (-0.08, 0.07) |
|  |  | -0.01 (-0.09, 0.06) |  |  | -0.01 (-0.15, 0.14) |  | |  | 0.02 (-0.06, 0.10) |  |  | -0.03 (-0.10, 0.04) |
| Fat mass index (kg/m^2^) | 0.059 | 0.06 (-0.08, 0.20) |  | 0.745 | -0.03 (-0.14, 0.09) |  | | 0.587 | -0.01 (-0.15, 0.13) |  | 0.692 | -0.01 (-0.15, 0.13) |
|  |  | -0.14 (-0.29, 0.01) |  |  | 0.03 (-0.27, 0.32) |  | |  | -0.07 (-0.23, 0.09) |  |  | -0.05 (-0.19, 0.09) |
| Fat-free mass index (kg/m^2^) | 0.736 | 0.02 (-0.06, 0.10) |  | 0.030 | 0.03 (-0.03, 0.10) |  | | 0.082 | -0.05 (-0.13, 0.04) |  | 0.785 | 0.02 (-0.07, 0.10) |
|  |  | 0.001 (-0.09, 0.09) |  |  | -0.17 (-0.35, -0.001) |  | |  | 0.06 (-0.03, 0.16) |  |  | -0.001 (-0.09, 0.09) |
| Weight-for-height (z-score) | 0.013 | 0.07 (-0.03, 0.16) |  | 0.504 | 0.002 (-0.07, 0.08) |  | | 0.782 | -0.03 (-0.13, 0.06) |  | 0.429 | 0.01 (-0.09, 0.11) |
|  |  | -0.11 (-0.21, -0.01) |  |  | -0.07 (-0.27, 0.13) |  | |  | -0.02 (-0.12, 0.10) |  |  | -0.05 (-0.14, 0.05) |
| Weight-for-age (z-score) | 0.029 | 0.02 (-0.05, 0.09) |  | 0.496 | -0.03 (-0.08, 0.02) |  | | 0.984 | -0.04 (-0.10, 0.03) |  | 0.428 | -0.01 (-0.08, 0.06) |
|  |  | -0.09 (-0.16, -0.02) |  |  | 0.02 (-0.12, 0.16) |  | |  | -0.05 (-0.11, 0.04) |  |  | -0.05 (-0.12, 0.02) |
| Mid-upper arm circumference (cm) | 0.902 | -0.02 (-0.13, 0.08) |  | 0.467 | -0.02 (-0.10, 0.06) |  | | 0.228 | -0.07 (-0.17, 0.03) |  | 0.470 | -0.06 (-0.16, 0.05) |
|  |  | -0.03 (-0.15, 0.08) |  |  | 0.06 (-0.15, 0.27) |  | |  | 0.03 (-0.09, 0.14) |  |  | -0.003 (-0.11, 0.10) |
| Triceps skinfold (mm) | 0.806 | 0.02 (-0.21, 0.26) |  | 0.278 | 0.06 (-0.12, 0.25) |  | | 0.593 | -0.05 (-0.28, 0.18) |  | 0.851 | 0.02 (-0.22, 0.26) |
|  |  | -0.02 (-0.28, 0.24) |  |  | -0.23 (-0.72, 0.26) |  | |  | 0.04 (-0.22, 0.31) |  |  | -0.02 (-0.26, 0.23) |
| Subscapular skinfold (mm) | 0.095 | 0.12 (-0.10, 0.33) |  | 0.856 | 0.002 (-0.17, 0.17) |  | | 0.331 | -0.08 (-0.29, 0.13) |  | 0.415 | -0.07 (-0.29, 0.15) |
|  |  | -0.15 (-0.38, 0.08) |  |  | -0.04 (-0.48, 0.40) |  | |  | 0.08 (-0.16, 0.32) |  |  | 0.06 (-0.16, 0.28) |
| Insulin-like growth factor-1 (ng/ml) | 0.652 | -0.76 (-4.63, 3.10) |  | 0.787 | 0.22 (-2.83, 3.28) |  | | 0.063 | 2.44 (-1.33, 6.20) |  | 0.922 | 0.21 (-3.83, 4.27) |
|  |  | -0.56 (-3.66, 4.77) |  |  | 1.42 (-6.73, 9.58) |  | |  | -3.03 (-7.38, 1.32) |  |  | -0.75 (-4.14, 3.99) |
| ^1^ Based on linear mixed effect models without adjustments. | | | | | | | | | | | | |
