# Supplementary material for: Effect of milk protein and whey permeate in large quantity lipid-based nutrient supplement on linear growth and body composition among stunted children: A randomized 2 × 2 factorial trial in Uganda
Source: PLoS Med. 2023 May 23;20(5):e1004227. doi: 10.1371/journal.pmed.1004227 (PMC10204948; doi:10.1371/journal.pmed.1004227)
Supplement: S4 Table — Adjusted and unadjusted analyses. (DOCX) [file pmed.1004227.s004.docx]

| S4 Table: Adjusted analysis: Subgroup effects of lipid-based nutrient supplement on growth by sex, breastfeeding status, stunting severity and inflammation among 750 children with stunting who received lipid-based nutrient supplement (n=600) or no supplement (n=150). Data shown are p for interaction and stratum-specific effect estimates (95% confidence interval) ^1^ | | | | | | | | | | | | |
| --- | --- | --- | --- | --- | --- | --- | --- | --- | --- | --- | --- | --- |
| Outcome | **Sex** | |  | **Breastfeeding** | |  | **Stunting severity** | | |  | **Inflammation** | |
|  | Boy (n=412) | |  | Not breastfeeding (n=651) | |  | Moderate (n=436) | | |  | No inflammation (n=270) | |
|  | Girl (n=338) | |  | Breastfeeding (n=95) | |  | Severe (n=314) | | |  | Inflammation (n=471) | |
|  | **Interaction, p** | **B (95% CI)** |  | **Interaction, p** | **B (95% CI)** |  | | **Interaction, p** | **B (95% CI)** |  | **Interaction, p** | **B (95% CI)** |
| Height (cm) | 0.794 | 0.55 (0.35, 0.74) |  | 0.512 | 0.57 (0.42, 0.72) |  | | 0.021 | 0.44 (0.26, 0.62) |  | 0.444 | 0.51 (0.31, 0.70) |
|  |  | 0.58 (0.37, 0.80) |  |  | 0.42 (-0.02, 0.86) |  | |  | 0.78 (0.55, 1.01) |  |  | 0.62 (0.42, 0.82) |
| Knee-heel length (mm) | 0.634 | 1.8 (1.1, 2.5) |  | 0.163 | 2.0 (1.5, 2.6) |  | | 0.084 | 2.3 (1.6, 2.9) |  | 0.054 | 1.4 (0.7, 2.1) |
|  |  | 2.0 (1.2, 2.8) |  |  | 0.9 (-0.7, 2.4) |  | |  | 1.3 (0.5, 2.2) |  |  | 2.4 (1.7, 3.1) |
| Height-for-age (z-score) | 0.806 | 0.17 (0.12, 0.23) |  | 0.505 | 0.17 (0.12, 0.21) |  | | 0.002 | 0.12 (0.07, 0.17) |  | 0.180 | 0.14 (0.08, 0.20) |
|  |  | 0.16 (0.10, 0.23) |  |  | 0.21 (0.08, 0.34) |  | |  | 0.25 (0.18, 0.32) |  |  | 0.20 (0.14, 0.26) |
| Weight (kg) | 0.711 | 0.20 (0.10, 0.29) |  | 0.063 | 0.24 (0.16, 0.31) |  | | 0.266 | 0.24 (0.15, 0.33) |  | 0.990 | 0.20 (0.11, 0.30) |
|  |  | 0.23 (0.12, 0.33) |  |  | 0.02 (-0.20, 0.24) |  | |  | 0.16 (0.04, 0.27) |  |  | 0.20 (0.11, 0.30) |
| Fat mass (kg) | 0.606 | 0.06 (-0.04, 0.16) |  | 0.192 | 0.06 (-0.02, 0.14) |  | | 0.223 | 0.08 (-0.02, 0.17) |  | 0.967 | 0.04 (-0.06, 0.14) |
|  |  | 0.02 (-0.09, 0.13) |  |  | -0.10 (-0.33, 0.13) |  | |  | -0.02 (-0.13, 0.10) |  |  | 0.04 (-0.03, 0.14) |
| Fat-free mass (kg) | 0.226 | 0.13 (0.06, 0.21) |  | 0.692 | 0.17 (0.11, 0.23) |  | | 0.800 | 0.16 (0.10, 0.23) |  | 0.858 | 0.15 (0.07, 0.24) |
|  |  | 0.20 (0.12, 0.28) |  |  | 0.13 (-0.04, 0.30) |  | |  | 0.18 (0.09, 0.26) |  |  | 0.16 (0.10, 0.23) |
| Fat mass index (kg/m^2^) | 0.712 | 0.03 (-0.11, 0.18) |  | 0.130 | 0.05 (-0.07, 0.16) |  | | 0.115 | 0.08 (-0.06, 0.22) |  | 0.961 | 0.01 (-0.14, 0.16) |
|  |  | -0.01 (-0.17, 0.16) |  |  | -0.23 (-0.57, 0.11) |  | |  | -0.10 (-0.28, 0.07) |  |  | 0.01 (-0.14, 0.16) |
| Fat-free mass index (kg/m^2^) | 0.103 | 0.02 (-0.07, 0.11) |  | 0.984 | 0.07 (0.001, 0.14) |  | | 0.108 | 0.11 (0.02, 0.19) |  | 0.724 | 0.07 (-0.03, 0.18) |
|  |  | 0.13 (0.03, 0.23) |  |  | 0.07 (-0.14, 0.28) |  | |  | -0.01 (-0.11, 0.10) |  |  | 0.05 (-0.03, 0.13) |
| Weight-for-height (z-score) | 0.892 | 0.08 (-0.02, 0.18) |  | 0.061 | 0.11 (0.03, 0.20) |  | | 0.013 | 0.16 (0.06, 0.25) |  | 0.871 | 0.08 (-0.02, 0.19) |
|  |  | 0.09 (-0.03, 0.21) |  |  | -0.13 (-0.37, 0.11) |  | |  | -0.05 (-0.17, 0.08) |  |  | 0.07 (-0.04, 0.18) |
| Weight-for-age (z-score) | 0.727 | 0.14 (0.07, 0.22) |  | 0.052 | 0.17 (0.11, 0.23) |  | | 0.248 | 0.17 (0.11, 0.24) |  | 0.888 | 0.14 (0.07, 0.22) |
|  |  | 0.16 (0.08, 0.24) |  |  | -0.003 (-0.17, 0.16) |  | |  | 0.11 (0.02, 0.20) |  |  | 0.15 (0.07, 0.23) |
| Mid-upper arm circumference (cm) | 0.292 | 0.09 (-0.02, 0.20) |  | 0.028 | 0.17 (0.08, 0.26) |  | | 0.109 | 0.18 (0.08, 0.29) |  | 0.174 | 0.07 (-0.04, 0.18) |
|  |  | 0.18 (0.06, 0.31) |  |  | -0.13 (-0.38, 0.12) |  | |  | 0.05 (-0.09, 0.18) |  |  | 0.18 (0.07, 0.30) |
| Triceps skinfold (mm) | 0.207 | -0.03 (-0.27, 0.21) |  | 0.515 | 0.10 (-0.09, 0.29) |  | | 0.115 | 0.18 (-0.05, 0.41) |  | 0.153 | 0.21 (-0.04, 0.46) |
|  |  | 0.21 (-0.06, 0.48) |  |  | -0.09 (-0.65, 0.46) |  | |  | -0.12 (-0.40, 0.17) |  |  | -0.05 (-0.30, 0.20) |
| Subscapular skinfold (mm) | 0.597 | -0.09 (-0.32, 0.14) |  | 0.474 | -0.07 (-0.25, 0.11) |  | | 0.084 | 0.06 (-0.16, 0.27) |  | 0.101 | 0.08 (-0.15, 0.32) |
|  |  | 0.003 (-0.25, 0.26) |  |  | 0.13 (-0.39, 0.66) |  | |  | -0.25 (-0.52, 0.02) |  |  | -0.20 (-0.44, 0.04) |
| Insulin-like growth factor-1 (ng/ml) | 0.531 | 5.04 (1.05, 9.04) |  | 0.068 | 5.43 (2.33, 8.53) |  | | 0.362 | 5.43 (1.67, 9.19) |  | 0.860 | 4.42 (0.29, 8.54) |
|  |  | 3.13 (-1.28, 7.55) |  |  | -3.66 (-12.90, 5.59) |  | |  | 2.60 (-2.15, 7.36) |  |  | 3.88 (-0.36, 8.13) |
| ^1^ Based on linear mixed effect models adjusted for age, sex, season and site. | | | | | | | | | | | | |

| S4 Table: Unadjusted: Subgroup effects of lipid-based nutrient supplement on growth by sex, breastfeeding status, stunting severity and inflammation among 750 children with stunting who received lipid-based nutrient supplement (n=600) or no supplement (n=150). Data shown are p for interaction and stratum-specific effect estimates (95% confidence interval) ^1^ | | | | | | | | | | | | |
| --- | --- | --- | --- | --- | --- | --- | --- | --- | --- | --- | --- | --- |
| Outcome | **Sex** | |  | **Breastfeeding** | |  | **Stunting severity** | | |  | **Inflammation** | |
|  | Boy (n=412) | |  | Not breastfeeding (n=651) | |  | Moderate (n=436) | | |  | No inflammation (n=270) | |
|  | Girl (n=338) | |  | Breastfeeding (n=95) | |  | Severe (n=314) | | |  | Inflammation (n=471) | |
|  | **Interaction, p** | **B (95% CI)** |  | **Interaction, p** | **B (95% CI)** |  | | **Interaction, p** | **B (95% CI)** |  | **Interaction, p** | **B (95% CI)** |
| Height (cm) | 0.702 | 0.54 (0.35, 0.73) |  | 0.508 | 0.58 (0.42, 0.73) |  | | 0.023 | 0.44 (0.25, 0.62) |  | 0.460 | 0.51 (0.31, 0.71) |
|  |  | 0.59 (0.38, 0.81) |  |  | 0.42 (-0.02, 0.86) |  | |  | 0.77 (0.54, 1.00) |  |  | 0.62 (0.41, 0.82) |
| Knee-heel length (mm) | 0.737 | 1.8 (1.1, 2.5) |  | 0.220 | 2.0 (1.5, 2.6) |  | | 0.091 | 2.2 (1.5, 2.9) |  | 0.055 | 1.4 (0.6, 2.1) |
|  |  | 2.0 (1.2, 2.8) |  |  | 1.0 (-0.7, 2.6) |  | |  | 1.3 (0.4, 2.1) |  |  | 2.4 (1.6, 3.1) |
| Height-for-age (z-score) | 0.867 | 0.17 (0.12, 0.23) |  | 0.469 | 0.16 (0.12, 0.21) |  | | 0.003 | 0.12 (0.07, 0.18) |  | 0.186 | 0.14 (0.08, 0.20) |
|  |  | 0.17 (0.10, 0.23) |  |  | 0.21 (0.09, 0.34) |  | |  | 0.25 (0.18, 0.32) |  |  | 0.20 (0.14, 0.26) |
| Weight (kg) | 0.806 | 0.20 (0.11, 0.30) |  | 0.036 | 0.24 (0.16, 0.31) |  | | 0.307 | 0.23 (0.14, 0.32) |  | 0.978 | 0.20 (0.10, 0.30) |
|  |  | 0.22 (0.11, 0.33) |  |  | -0.01 (-0.23, 0.21) |  | |  | 0.16 (0.04, 0.27) |  |  | 0.20 (0.10, 0.30) |
| Fat mass (kg) | 0.699 | 0.06 (-0.04, 0.16) |  | 0.212 | 0.06 (-0.02, 0.14) |  | | 0.261 | 0.08 (-0.02, 0.17) |  | 0.921 | 0.04 (-0.06, 0.15) |
|  |  | 0.03 (-0.09, 0.14) |  |  | -0.10 (-0.33, 0.14) |  | |  | -0.01 (-0.13, 0.11) |  |  | 0.06 (-0.07, 0.14) |
| Fat-free mass (kg) | 0.410 | 0.14 (0.06, 0.21) |  | 0.465 | 0.17 (0.11, 0.23) |  | | 0.755 | 0.15 (0.08, 0.22) |  | 0.939 | 0.15 (0.07, 0.23) |
|  |  | 0.19 (0.10, 0.27) |  |  | 0.10 (-0.07, 0.27) |  | |  | 0.17 (0.08, 0.26) |  |  | 0.16 (0.08, 0.24) |
| Fat mass index (kg/m^2^) | 0.709 | 0.04 (-0.11, 0.19) |  | 0.113 | 0.05 (-0.07, 0.17) |  | | 0.125 | 0.08 (-0.06, 0.23) |  | 0.801 | 0.03 (-0.13, 0.18) |
|  |  | -0.01 (-0.18, 0.17) |  |  | -0.25 (-0.61, 0.10) |  | |  | -0.10 (-0.28, 0.08) |  |  | -0.001 (-0.15, 0.15) |
| Fat-free mass index (kg/m^2^) | 0.122 | 0.02 (-0.07, 0.11) |  | 0.934 | 0.06 (-0.01, 0.13) |  | | 0.179 | 0.09 (0.01, 0.18) |  | 0.929 | 0.05 (-0.04, 0.15) |
|  |  | 0.12 (0.02, 0.23) |  |  | 0.05 (-0.16, 0.27) |  | |  | -0.004 (-0.11, 0.10) |  |  | 0.06 (-0.04, 0.15) |
| Weight-for-height (z-score) | 0.924 | 0.08 (-0.02, 0.18) |  | 0.042 | 0.11 (0.03, 0.20) |  | | 0.017 | 0.15 (0.05, 0.25) |  | 0.928 | 0.08 (-0.03, 0.19) |
|  |  | 0.09 (-0.03, 0.21) |  |  | -0.15 (-0.39, 0.09) |  | |  | -0.04 (-0.17, 0.08) |  |  | 0.07 (-0.04, 0.18) |
| Weight-for-age (z-score) | 0.781 | 0.14 (0.07, 0.22) |  | 0.036 | 0.17 (0.11, 0.23) |  | | 0.259 | 0.17 (0.10, 0.24) |  | 0.888 | 0.14 (0.07, 0.22) |
|  |  | 0.16 (0.08, 0.24) |  |  | -0.02 (-0.18, 0.15) |  | |  | 0.11 (0.02, 0.20) |  |  | 0.15 (0.07, 0.23) |
| Mid-upper arm circumference (cm) | 0.384 | 0.10 (-0.01, 0.21) |  | 0.028 | 0.17 (0.08, 0.26) |  | | 0.104 | 0.18 (0.08, 0.29) |  | 0.163 | 0.07 (-0.05, 0.18) |
|  |  | 0.17 (0.05, 0.30) |  |  | -0.13 (-0.38, 0.12) |  | |  | 0.04 (-0.09, 0.17) |  |  | 0.18 (0.07, 0.30) |
| Triceps skinfold (mm) | 0.141 | -0.05 (-0.30, 0.21) |  | 0.345 | 0.11 (-0.09, 0.31) |  | | 0.222 | 0.16 (-0.08, 0.40) |  | 0.227 | 0.19 (-0.06, 0.45) |
|  |  | 0.24 (-0.04, 0.52) |  |  | -0.18 (-0.76, 0.40) |  | |  | -0.08 (-0.39, 0.22) |  |  | -0.03 (-0.29, 0.23) |
| Subscapular skinfold (mm) | 0.625 | -0.08 (-0.32, 0.15) |  | 0.661 | -0.06 (-0.25, 0.12) |  | | 0.074 | 0.07 (-0.15, 0.28) |  | 0.109 | 0.08 (-0.15, 0.32) |
|  |  | 0.002 (-0.26, 0.26) |  |  | 0.06 (-0.47, 0.59) |  | |  | -0.25 (-0.53, 0.02) |  |  | -0.19 (-0.43, 0.05) |
| Insulin-like growth factor-1 (ng/ml) | 0.718 | 4.38 (0.29, 8.48) |  | 0.048 | 5.32 (2.11, 8.52) |  | | 0.635 | 4.63 (0.74, 8.51) |  | 0.941 | 3.93 (-0.32, 8.18) |
|  |  | 3.26 (-1.27, 7.78) |  |  | -4.76 (-14.21, 4.70) |  | |  | 3.11 (-1.81, 8.03) |  |  | 3.70 (-0.68, 8.08) |
| ^1^ Based on linear mixed effect models without adjustments. | | | | | | | | | | | | |
