# Supplementary material for: Effect of milk protein and whey permeate in large quantity lipid-based nutrient supplement on linear growth and body composition among stunted children: A randomized 2 × 2 factorial trial in Uganda
Source: PLoS Med. 2023 May 23;20(5):e1004227. doi: 10.1371/journal.pmed.1004227 (PMC10204948; doi:10.1371/journal.pmed.1004227)
Supplement: S1 Text — (DOCX) [file pmed.1004227.s006.docx]

Research Protocol

The role of milk protein and whey permeate in the growth and development of stunted children: a randomised controlled trial in Eastern Uganda.

Study acronym: MAGNUS – Milk affecting growth, cognition and the gut in child stunting

Study ID number: D222

Protocol version: 05

Date: 03-12-2019

# Administrative information

| Title | The role of milk protein and whey permeate in the growth and development of stunted children: a randomised controlled trial in Eastern Uganda. |
| --- | --- |
| Version | V05-03-12-2019 |
| Study site | Busoga subregion, Eastern Uganda |
| Funding | 1) Arla Food for Health (a centre for dairy research between Arla Foods amba, Arla Foods Ingredients Group P/S, Aarhus University and University of Copenhagen), 2) Danish Dairy Research Foundation, 3) Department of Nutrition, Exercise and Sports, University of Copenhagen, Denmark |
| Investigational product development | Nutriset, France |
| Study design | Randomised controlled trial |
| Trial sponsor | Department of Nutrition, Exercise and Sports, University of Copenhagen, Denmark |
| Sponsor representative | Henrik Friis, Professor, MD, PhD, Department of Nutrition, Exercise and Sports, University of Copenhagen, Denmark |
| Principal investigators | 1) Ezekiel Mupere, PhD, Pediatrician, Epidemiologist, Head of Department of Paediatrics and Child Health, Makerere University, Uganda 2) Henrik Friis, Professor, MD, PhD, Department of Nutrition, Exercise and Sports, University of Copenhagen, Denmark |
| Principal co-investigator | Benedikte Grenov, Postdoc, PhD, MSc, Department of Nutrition, Exercise and Sports, University of Copenhagen, Denmark |
| Co-Investigators | 1) Christian Mølgaard, Professor, MD, PhD, Head of Section^1^ 2) Kim Fleischer Michaelsen, Prof, MD, DMSc^1^ 3) Suzanne Filteau, Prof, MSc, PhD, 4) André Briend, Affiliate professor, MD, PhD^1, 4^ 5) Mette Frahm Olsen, Postdoc, PhD, MSc^1^ 6) Joseph Mbabazi, PhD student, MSc^1,2^ 7) Rolland Mutumba, PhD student, MD^1,2^ 8) Hannah Pesu, PhD student, MSc^1^ |
| University affiliations | 1: Department of Nutrition, Exercise and Sports, University of Copenhagen, Denmark. 2: Department of Paediatrics and Child Health, Makerere University, Uganda. 3: London School of Hygiene and Tropical Medicine, 4: University of Tampere, Finland. |
| Conflict of interest | The principal investigators declare that they have no financial interest in the results of this study. Neither Arla Foods amba, Arla Foods Ingredients Group nor the Danish Dairy Research Foundation have any influence on the reporting of study results. |
| Trial Registration | The study will be registered at <http://www.isrctn.com/> when IRB approval has been obtained and before enrolment of any participants. |

# Study investigators and agreements

## Signatures and agreement with protocol

We, the undersigned, acknowledge that we have read this protocol. We agree to conduct this study in accordance with the study protocol, the current version of the Declaration of Helsinki, and with any additional local laws and regulations.

| Principal Investigator/Sponsor representative  ____________________________________________  Henrik Friis, Professor, MD, PhD, Department of Nutrition, Exercise and Sports, University of Copenhagen, Denmark | Principal Investigator      ____________________________________________  Ezekiel Mupere, PhD, Paediatrician, Epidemiologist, Head of Department of Paediatrics and Child Health, Makerere University |
| --- | --- |
| Principal co-investigator  ___________________________________________  Benedikte Grenov, Postdoc, PhD, MSc, Department of Nutrition, Exercise and Sports, University of Copenhagen, Denmark | Co-investigator  _______________________________________  Kim Fleischer Michaelsen, Prof, MD, DMSc, Department of Nutrition, Exercise and Sports, University of Copenhagen, Denmark |
| Co-investigator  __________________________________________  Christian Mølgaard, Professor, MD, PhD, Head of Section, Department of Nutrition, Exercise and Sports, University of Copenhagen, Denmark | Co-investigator  ____________________________________________  Joseph Mbabazi, PhD student, MSc, Makerere University Uganda |
| Co-investigator  ___________________________________________  André Briend, Affiliate professor, MD, PhD, Department of Nutrition, Exercise and Sports, University of Copenhagen, Denmark and the University of Tampere, Finland | Co-investigator  ___________________________________________  Rolland Mutumba, PhD student, Paediatrician, Makerere University Uganda |
| Co-investigator  ___________________________________________  Suzanne Filteau, Professor, MSc, PhD, Department of Population Health, London School of Hygiene and Tropical Medicine | Co-investigator  ________________________________________  Mette Frahm Olsen, Postdoc, PhD, MSc, Department of Nutrition, Exercise and Sports, University of Copenhagen, Denmark |
| Co-investigator  ____________________________________________  Hannah Pesu, PhD student, MSc, Department of Nutrition, Exercise and Sports, University of Copenhagen, Denmark |  |

# Abbreviations

| CDI | Child development index |
| --- | --- |
| CRF | Case report form (data collection document) |
| CRF | Case report form |
| GCP | Good Clinical Practice |
| HAZ | Height-for-age Z-score |
| HSP | Human Subject Protection |
| IRB | Institutional Review Board |
| ITC | Inpatient clinic – for the treatment of complicated SAM |
| LNS-LQ | Large-quantity lipid-based nutrient supplements |
| MAGNUS | Milk affecting growth, cognition and the gut in child stunting |
| MDAT | Malawi development assessment tool |
| MPI | Milk protein isolate |
| MUAC | Mid-upper-arm circumference |
| OTC | Outpatient clinic - for the treatment of uncomplicated SAM |
| SAE | Serious adverse event |
| SAM | Severe acute malnutrition |
| SOP | Standard operating procedures |
| SPI | Soy protein isolate |
| UNCST | The Ugandan National Council of Science and Technology |
| VHTs | Village health teams (refers to one or several people) |
| EED | Environmental enteric dysfunction |
| WAZ | Weight-for-age z-score |
| WHZ | Weight-for-height z-score |
| WP | Whey permeate |

# Definitions of terms

| Stunting | Also known as linear growth faltering or short-for-age. Defined as height-for-age Z-score < -2. |
| --- | --- |
| Milk protein isolate (MPI): | A milk ingredient obtained by isolating the protein components of milk (casein and whey proteins). Milk protein isolate contains a minimum of 90% milk proteins. |
| Lipid based nutrient supplements (LNS): | A generic term for fortified, lipid-based ready-to-use supplementary or therapeutic products which are modified in their energy density, protein, fat or micronutrient composition to help meet the nutritional requirements of specific populations. They are currently in use in the treatment or prevention of child malnutrition. |
| Whey permeate (WP): | A product of whey ultrafiltration. Proteins are removed, contains over 80/85% lactose and milk minerals. |
| Lactose | A disaccharide which is the main carbohydrate component of human breast milk (approx. 7%) and makes up approximately 4.8% of cow’s milk. |
| Milk minerals | Highly bioavailable minerals found in milk; potassium, phosphorus, magnesium, calcium, sodium and to a lesser extent zinc. |
| Environmental enteric dysfunction | A generalised inflammatory disorder of the small intestine that is thought to be widespread among children living in low-income country settings. |

# Table of contents

[1 Administrative information 2](#_Toc26278885)

[2 Study investigators and agreements 3](#_Toc26278886)

[3 List of responsible parties 4](#_Toc26278887)

[4 Abbreviations 5](#_Toc26278888)

[5 Definitions of terms 6](#_Toc26278889)

[6 Table of contents 7](#_Toc26278890)

[7 Summary of protocol 8](#_Toc26278891)

[8 Introduction 11](#_Toc26278892)

[9 Objectives 13](#_Toc26278893)

[10 Trial design 14](#_Toc26278894)

[11 Study setting and participants 17](#_Toc26278895)

[12 Intervention 18](#_Toc26278896)

[13 Reference group 21](#_Toc26278897)

[14 Deterioration and referral 21](#_Toc26278898)

[15 Concomitant care for study participants 21](#_Toc26278899)

[16 Strategies to improve and monitor adherence 21](#_Toc26278900)

[17 Outcomes 22](#_Toc26278901)

[18 Sample size 24](#_Toc26278902)

[19 Schedule of measurements and assessment 25](#_Toc26278903)

[20 Assignment of interventions 25](#_Toc26278904)

[21 Pilot phase 26](#_Toc26278905)

[22 Data collection and measurements 27](#_Toc26278906)

[23 Quality assurance 32](#_Toc26278907)

[24 Data management 32](#_Toc26278908)

[25 Statistical methods 33](#_Toc26278909)

[26 Assessing and reporting adverse events and reactions 33](#_Toc26278910)

[27 Monitoring 34](#_Toc26278911)

[28 Ethics and dissemination 35](#_Toc26278912)

[29 References 39](#_Toc26278913)

[30 Appendices 42](#_Toc26278914)

# Summary of protocol

| **Title of Study:**  The role of milk protein and whey permeate in the growth and development of stunted children. | |
| --- | --- |
| **Acronym:**  MAGNUS – Milk affecting growth, cognition and the gut in child stunting | |
| **Study Code:**  D-222 | |
| **Funding:**  1) Arla Food for Health 2) Danish Dairy Research Foundation, 3) University of Copenhagen, Department of Nutrition, Exercise and Sports. | |
| **Sponsor:**  University of Copenhagen, Department of Nutrition, Exercise and Sports  **Sponsor representative:**  Henrik Friis, Professor, MD, PhD, University of Copenhagen, Section for Paediatrics and International Nutrition, Denmark | |
| **Principal Investigators:**  1) Ezekiel Mupere, PhD, Pediatrician, Epidemiologist, Head of Department of Paediatrics and Child Health, Makerere University, Uganda 2) Henrik Friis, Professor, MD, PhD, University of Copenhagen, Department of Nutrition, Exercise and Sports, Denmark | |
| **Study Operations:**  Study Site: Site operations facilitated from two health centres in Jinja. Agreements with local government offices in Jinja and Mayuge districts to expand if further sites are needed. Central office in Jinja town. | |
| **Planned Study Period:**  First patient in: January 2020, Last patient out: June 2021 | |
| **Objectives:**  The primary objective is to assess the effects of supplementation with milk protein isolate (MPI) and whey permeate (WP) on linear growth in stunted children.  Secondary objectives are to assess the effects of supplementation with MPI and WP on child development, on anthropometric measurements, on body composition and blood haemoglobin concentration in stunted children. | |
| **Measurable Outcomes:**  Primary outcome: Knee-heel length (mm) and total length (cm) over 12 weeks.  Secondary outcomes: HAZ, WAZ, WHZ, weight gain (g), body composition (bioimpedance and skin folds), head circumference, MUAC, haemoglobin (Hb) and child development, based on the Malawi Developmental Assessment Tool (MDAT) over 12 weeks.  Tertiary outcomes: Growth factors [Insulin-like growth factor-1 (IGF-1), insulin], markers of nutrient status [ferritin, soluble transferrin receptor (sTfR), vitamin B12, vitamin A and folate], markers of systemic inflammation, intestinal inflammation and intestinal function [C-reactive protein (CRP), alpha-1-acid glycoprotein (AGP), myeloperoxidase (MPO), neopterin (NEO), alpha-1-antitrypsin (AAT), citrulline], gut microbiota (diversity and composition), and morbidity over 12 weeks. | |
| **Study participants:**  750 children aged 12 -59 months with a HAZ <-2. | |
| **Study design:**  A randomised, double-blinded, community trial, testing four different large-quantity lipid-based nutrient supplements (LNS-LQ) in a two-by-two factorial design. A fifth un-supplemented group is included as a reference. Shown in figure 1. | |
| \|  \|  \| + Milk protein isolate (MPI) \|  \| + Soy protein Isolate (SPI) \| \| --- \| --- \| --- \| --- \| --- \| \| Whey permeate  (+ WP) \| A. \| LNS-LQ + MPI + WP  n=150 \| C. \| LNS-LQ + SPI + WP  n=150 \| \| No whey permeate  (-WP) \| B. \| LNS-LQ + MPI – WP  n=150 \| D. \| LNS-LQ + SPI - WP  n=150 \|  \|  \| Family diet \| \| --- \| --- \| \| E. \| Reference n=150 \|   **Figure 1:** Four large-quantity lipid-based nutrient supplements (LNS-LQ) in a two-by-two factorial design. Reference group continues with family diet. n= number of children allocated. MPI: Milk protein isolate, WP: whey permeate, SPI: soy protein isolate | |
| **Study site and recruitment:**  Study clinics are run from selected community health centres. Recruitment will take place in villages located nearby these selected community health centers | |
| **Participant inclusion:**  If both inclusion and exclusion criteria are fulfilled and informed consent is given, children are randomly allocated to one of four LNS-LQ or to an un-supplemented group. | |
| **Criteria for inclusion:**   - Age 12 - 59 months - HAZ < -2 according to WHO growth standards (2006) - Care-taker able and willing to return for follow-up visits and agrees to phone follow-up - Living within the catchment area - Written informed consent given by parent/caregiver   **Criteria for exclusion:**   - SAM, defined as MUAC <115 mm OR WHZ<-3 OR bipedal pitting oedema - Medical complications requiring hospitalization. - Obvious disability that impedes eating capacity - Disability that makes length/height assessment problematic - Participation in another study which impacts on this study or previous enrollment in this study. - Family plans to move away from the catchment area in the next 6 months. - History or known allergy to peanuts or milk | |
| **Intervention:**  A supplementary food intervention. Of the 750 children included in the study, 600 are randomly allocated to one of four LNS-LQ upon inclusion and 150 are randomly allocated to no supplementation. All children receive nutrition counselling at inclusion. Those randomised to LNS are given one sachet of 100g per day in addition to the home based diet. Each 100g LNS-LQ sachet provides approximately 530kcal, provides up to half of the average daily energy requirements, and satisfies most of the estimated daily micronutrient requirements (age dependent). | |
| **Duration of intervention:**  12 week intervention. | |
| **Data collection and follow-up**  **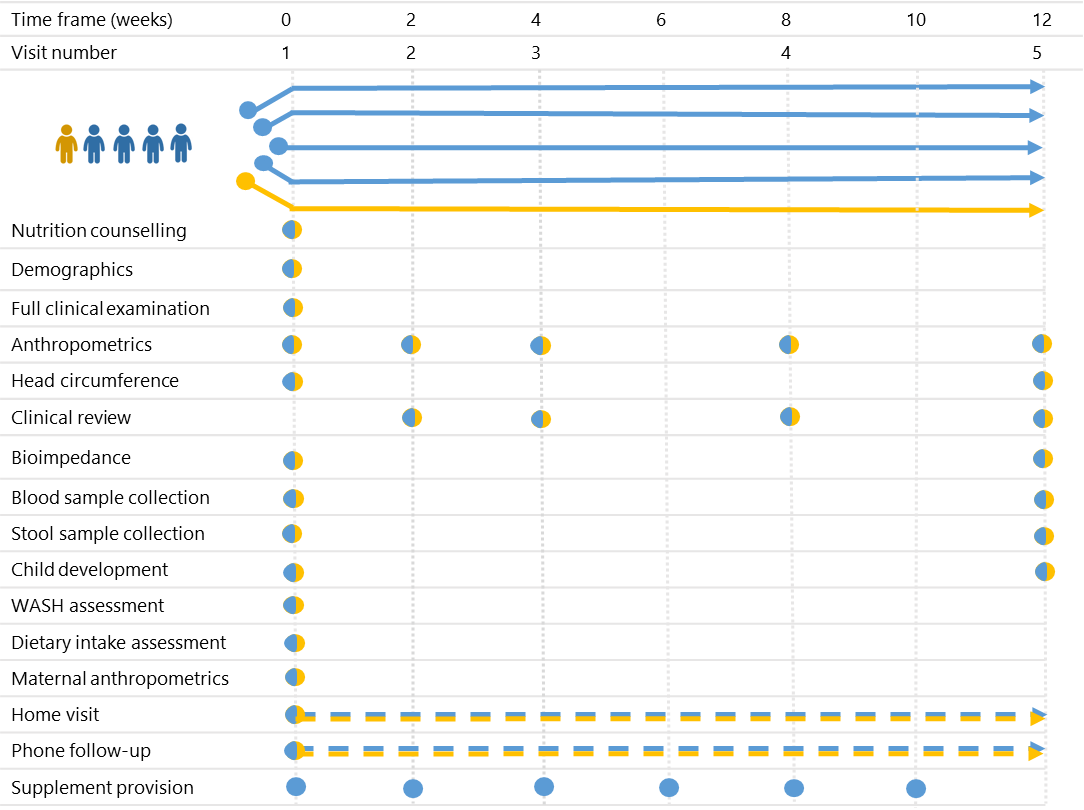** | |
| **Figure 2:** Study design framework and time-points for data collection.  The 750 children with a HAZ <-2 are included and randomly allocated to one of four LNS-LQ or to a reference group. Supplemented children are provided one LNS-LQ sachet per day (in two-week allocations) over a period of 12 weeks. After inclusion, all children, irrespective of allocation are followed-up every 2-4 weeks until 12 weeks. | **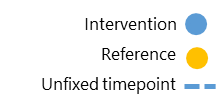** |
| **Statistical Methods:**  Primary, secondary, and tertiary endpoints are analysed by means of longitudinal data analysis since repeated measurements are recorded per patient. | |

# Introduction

## Background

Stunting is the most prevalent form of malnutrition: it is estimated to affect one in every fourth child, or 149 million children globally [1]. While acute malnutrition has been steadily in decline over the last decade, stunting is a particular challenge in East Africa, where according to the WHO, absolute numbers are on the rise due to population growth [1]. Stunting is defined as having a height-for-age Z score (HAZ) that is less than -2, according to the WHO growth standards [2]. Stunting has been associated with increased risk of mortality, impaired immune function, and delayed physical and cognitive development [3]. The knock on effects from stunting have the potential to reduce physical capacity and economic productivity later in life [4], and in this way, widespread stunting also has the potential to impact on societal economic development. In Uganda, nearly one in three children under five are stunted, which is similar to proportions reported in the Busoga sub-region in Eastern Central Uganda [5]. It is of fundamental importance to find the means to prevent stunting for future generations, but is arguably equally important to find solutions to improving outcomes for those children who are already stunted. While the pathogenesis of stunting remains unclear, several risk factors have been identified such as; foetal growth restriction, exposure to unsanitary environments and nutritionally inadequate diets throughout the early years of life [6]. Many of these factors have been the target of intervention in recent years.

It is well established that adequate nutrient intake in the early years plays a crucial role in growth and cognitive development. A nationwide analysis reported that most of the Busoga sub-region is entering either a stressed or a crisis phase of food insecurity [7], and this is likely to exacerbate nutrient deficiencies. Moderate to severe anaemia among children in the Busoga Sub-Region are reported to be over 40 percent, 10 percent above the national average [5]. Such nutrient deficiencies may be further exacerbated by the presence of a generalised inflammatory disorder of the small intestine which is thought to be widespread among children living in settings where they are frequently exposed to conditions of inadequate sanitation and hygiene [8]. This enteric state called environmental enteric dysfunction (EED), may impair the appropriate absorption and utilisation of nutrients. While this condition is considered reversible, it is thought to be an important contributor to the aetiology of stunting in early life. In settings where food insecurity is a concern and where nutrient deficiencies are widespread, specially formulated, lipid-based nutrient supplements (LNS) can be of great benefit in filling the nutrient gaps in the home diet [9].

Often LNS supplements contain milk products. One reason for this is that studies from both low and high income countries shown that milk may have a growth promoting effect [10] and has been associated with increases in lean mass deposition, and benefits to cognitive function, even beyond the early developmental years [11]. To better understand the possible beneficial effects of milk, consideration can be given to the different milk ingredients separately. The two major types of protein in milk are casein and whey. They are thought to promote growth by stimulating growth factors IGF-1 and insulin. Milk proteins have a rich amino acid (AA) profile, containing all essential AAs; both branched and sulphur containing, which are important for lean tissue deposition [12].

Whey permeate is the portion of milk that contains the lactose and naturally occurring minerals, each may contribute unique growth stimulating effects. Whey permeate is approximately 80-85 percent lactose and may also have a prebiotic effect, stimulating the growth of beneficial bacteria in the intestine [13]*.* The highly concentrated and bioavailable milk minerals (potassium, phosphorus, magnesium, calcium, sodium and to a lesser extent zinc), may also play a role in bone mineralisation and lean tissue deposition [14].

Non-milk sources of protein, such as soy have also been used in LNS to stimulate growth [15–17]. Soy is one of the most amino acid rich sources of plant protein available. Dehulled soy is the most common soy protein used in supplements. Due to minimal processing however, dehulled soy contains phytates which can reduce the bioavailability of essential micronutrients including calcium, zinc and iron, and may also hinder protein absorption [18]. On the other hand, soy protein isolate (SPI) is over 90 percent protein and has been processed to remove the phytate content so that SPI has a high Digestible Indispensable Amino Acid Score (DIAAS).

A number of studies aiming to prevent stunting have seen some positive effects on linear growth and haemoglobin levels after provision of small daily doses of LNS during the first 1000 days of life [19–22]. A recently published meta-analysis confirms that the use of LNS in combination with complementary feeding is a more effective means to improve such outcomes than no intervention [23]. Overall however, the benefit on linear growth from supplementation with small quantity LNS has been limited [24]. It may be that the amount of milk protein provided in a 20g sachet is not adequate to replenish nutrient deficiencies and stimulate growth. In order to provide the equivalent of one glass of milk per day in children older than one year, a larger quantity of LNS is necessary [25] .

Lipid based nutrient supplements (LNS) have been developed in an effort to bridge the nutrient gaps and to encourage growth and repair in young children. While the first 1000 days are extremely important, there is also reason to believe that interventions after this stage can have a measurable impact on growth and cognitive development [9].This study will provide new and unique perspectives on child stunting in several ways. First this study explores the effects of milk ingredients (milk proteins and whey permeate) on growth, child development, body composition, haemoglobin and a number of functional outcomes. Furthermore, we explore the use of large quantity LNS (LNS-LQ) to improve outcomes of stunted children aged 12-59 months. The findings from this study will improve our knowledge on the value of milk ingredients in the treatment of child stunting before and beyond the first 1000 days as well as contributing to the base of knowledge in order to better prevent stunting.

## Study location and capacity building

The study is located in the Busoga subregion of Eastern Uganda and will be implemented at community health clinics in collaboration with the Department of Paediatrics and Child Health, Makerere University, Kampala, Uganda. Decisions concerning where study sites should be located have been made in collaboration with the District Health Officer (DHO) and the acting responsible of relevant community health clinics. Two Ugandan PhD students are included as co-researchers on the team. The study also involves qualified local nurses, nutritionists, child development officers, lab technicians, village health workers (VHTs), and where possible, masters students.

# Objectives

## Overall study objective

To assess the effectiveness of milk protein isolate and whey permeate in improving growth and development in stunted Ugandan children aged 12-59 months.

## Primary objective

To assess the effects of supplementation with milk protein isolate and whey permeate on; linear growth based on knee-heel length and total length.

## Secondary objectives

To assess the effects of supplementation with milk protein isolate and whey permeate on child development, blood haemoglobin concentration, and on anthropometric measurements including; height-for-age Z-score, weight-for-height Z-score, weight-for-age Z-score, mid-upper arm circumference, skin folds and body composition.

# Trial design

## Overall study design and duration

This is a randomised, double-blinded, community-based controlled trial, which tests the effects of whey permeate and milk protein isolate on growth in stunted children aged 12-59 months in a two-by-two factorial design (Figure 1). All of the 750 children included in the study receive nutrition counselling at baseline, of these, 600 will be randomised to one of four supplements for a 12 week duration. As a reference, a further 150 children will be randomised to continue with the family diet for 12 weeks. All children are followed-up at regular intervals during these 12 weeks. (Figure 2).

|  |  | + Milk protein isolate (MPI) |  | + Soy protein Isolate (SPI) |
| --- | --- | --- | --- | --- |
| Whey permeate  (+ WP) | A. | LNS-LQ + MPI + WP  n=150 | C. | LNS-LQ + SPI + WP  n=150 |
| No whey permeate  (-WP) | B. | LNS-LQ + MPI – WP  n=150 | D. | LNS-LQ + SPI – WP  n=150 |
|  |  |  |  |  |
|  |  |  |  | Family diet |
|  |  |  | E. | Reference group n=150 |

**Figure 1: Four large-quantity lipid-based nutrient supplements (LNS-LQ) in a two-by-two factorial design** (A, B, C and D). Reference group (E) continues with family diet. n= number of children allocated, MPI: Milk protein isolate, WP: whey protein, SPI: soy protein isolate

No supplementation

Supplementation

**Inclusion**

**12 wks/ Discharge**

E. Reference n= 150

Children HAZ <-2

N = 750

A. LNS-LQ n= 150

B. LNS-LQ n= 150

C. LNS-LQ n= 150

D. LNS-LQ n= 150

**Figure 2: MAGNUS study design framework.** 750 children with HAZ <-2 are included and randomised to one of four supplements or to a reference group. LNS-LQ is allocated for a fixed duration of 12 weeks after inclusion.. The reference group is not supplemented. LNS-LQ: Large-quantity lipid-Based Nutrient Supplement. HAZ: Height-for-age Z score

## Description of study flow and processes

### Community mobilisation

Prior to recruitment within a particular community, community leaders will be informed about the study to strengthen community engagement in support of pre-screening for the study.

### Pre-screening and referral

Village health teams (VHTs) will mobilise communities and refer children to a local pre-screening site within the community. The pre-screening team will screen children for stunting and severe acute malnutrition (SAM). Those identified as stunted are referred according to the SOP. Children identified as having SAM are referred to outpatient care (OTC) or inpatient care (ITC) for relevant treatment. Pre-screening data will be recorded with a pre-screening ID. The VHTs will be remunerated for their work in the study.

### Screening

Screening is carried out at the study site. All children screened are allocated a screening ID number. Study staff who are trained in taking anthropometrics will measure the child’s MUAC, weight, height/length and check for oedema. If the child is classified as being stunted without SAM, qualified clinical study staff will assess the child according to the remaining inclusion and exclusion criteria. If all criteria are met, the caregiver is taken through the informed consent process. If informed consent is given, the child is included in the study.

### Inclusion

Upon inclusion, children are allocated a unique study ID number and are taken through a registration process according to the relevant SOP.

### Baseline measurements

Following inclusion, baseline measurements are taken according to the relevant SOPs. An example of the site flow at baseline is provided in figure 3. The following measurements are taken/assessed at baseline: questionnaires of medical history, demographics and dietary information, clinical assessment is conducted, blood and stool samples are collected, anthropometrics of the mother (if available) and child are measured, bioimpedance and child development assessed. A full description of the data collected at baseline is provided in section 22.

### Nutrition counselling and training

All caregivers receive locally adapted nutrition counselling. If stool sample collections are not possible at the baseline clinic visit, the caregiver is given instruction and a kit for stool collection at home. Refer to the SOP for further detail.

**
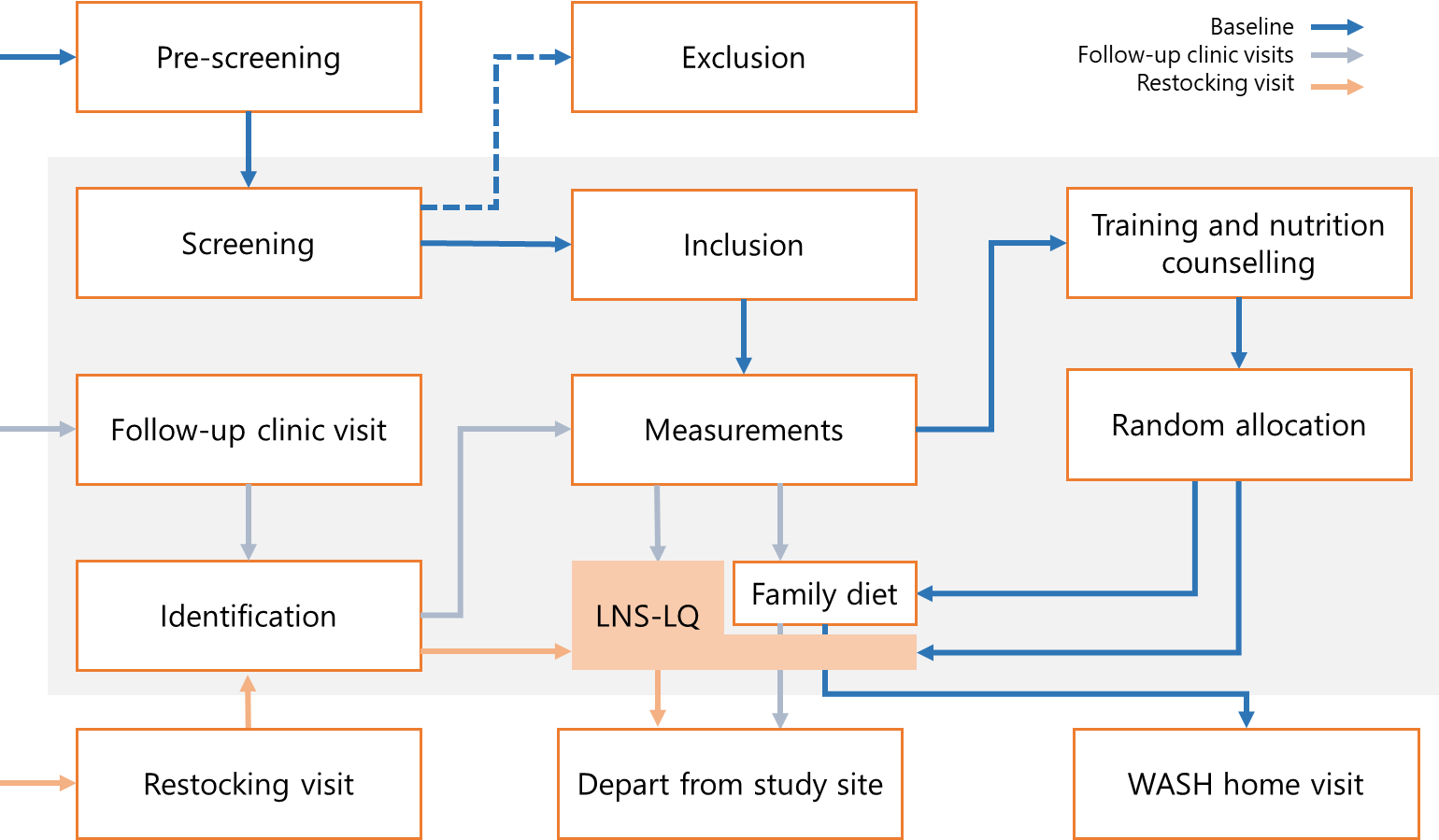
**

**Figure 3: Basic study site flow.** Includes flow at baseline and for follow-up clinic visits. This is only a visual representation and may be subject to change. The SOP gives further detail. Measurements include sample collection at baseline and week 12 visits. LNS-LQ: Large-quantity lipid-based nutrient supplements; WASH: water, sanitation and hygiene.

### Random allocation

Thereafter, caregivers are informed of allocation to supplementation or the reference group. Those not allocated to supplementation are given a token of appreciation (given at each follow-up clinic visit), valued at around 5000 UGX but not more than 10 000UGX, for example clothes soap. If allocated to LNS caregivers are given training concerning appropriate use of LNS before they are allocated a two week supply. Prior to departure all caregivers are given a follow-up plan. Refer to the SOP for further details.

### Follow-up clinic visits

All caregivers and study participants are requested to return to a specific study site for a follow-up visit at 2, 4, 8 and 12 weeks post inclusion. Those receiving LNS are also requested to return at 6 and 10 weeks post inclusion to restock sachets.

At follow-up clinics child anthropometrics are measured, a clinical review is done and inquiries are made into LNS compliance (if relevant). At week 12, additional parameters are measured; blood and stool samples are collected and bioimpedance and child development are assessed and a discharge questionnaire is completed. An overview of the measurements taken at each time point are provided in figure 4.

### Home visits and WASH

On the day of inclusion (or as close as possible to inclusion date), staff/s will follow each caregiver home and will map the GPS coordinates of the house. At the first home visit, trained study staff will make an assessment of water sanitation and hygiene (WASH) characteristics and record this in the CRF. The WASH assessment is further described in the related SOP. Home visits are necessary to ensure that information is collected in a timely manner from participants who miss study clinic visits. Home visits will be carried out by well-trained study staff.

### Phone follow-up

Phone follow-up will be attempted in order to encourage caregiver attendance at designated appointment times as well as to gather basic health and compliance information during the study period. See section 22.9 for more information.

# Study setting and participants

## Study setting

The study is based in the districts of Jinja and Mayuge in the Busoga sub-region, Eastern Uganda. Here, the prevalence of child stunting is 29 percent, and although high, this is similar to the national average in Uganda [5]. The study clinics are set up at local community health centers. Local health centers are a keystone in providing healthcare access to children living in both rural and urban communities. This study will be incorporated into the community health center framework and will train local staff where possible. Appendix 1 provides a list of eight community health centers for which an agreement has been reached with the district health officer in order that study clinic operations may be carried out at those locations. Recruitment will begin at two health centers in the district of Jinja. Activities will expand to other clinics only if participant recruitment is slow. The study office is based in Jinja.

## Study participants

Seven hundred and fifty (N=750) children aged 12 to 59 months with a height-for-age z-score of less than -2 who meet the eligibility criteria will be invited to participate in the study.

## Eligibility criteria

### Inclusion criteria

- Aged 12 - 59 months.
- HAZ <-2 (WHO growth standards).
- Care-taker able and willing to return for follow-up visits, an initial home visit and agrees to phone follow-up.
- Living within the catchment area.
- Written informed consent given by parent/caregiver.

### Exclusion criteria

- SAM; measured as MUAC < 115mm OR weight-for-height z-score < -3 OR bilateral pitting oedema
- Medical complications requiring hospitalization.
- Obvious disability that impedes eating capacity.
- Disability that makes length/height assessment problematic
- Participation in another study or program which impacts on this study or previously enrolled in this study.
- Family plans to move away from the catchment area in the next 6 months.
- History or known allergy to peanuts or milk.

# Intervention

This is a community-based supplementary food intervention.

## Description of LNS-LQ investigational products

In this study, four different LNS-LQ formulations are used to supplement the diet of stunted children. The supplements are composed in a two by two factorial design, varying with respect to the incorporation of milk based ingredients; whey permeate and milk protein isolate. These supplements are standardized to contain similar proportions of energy, protein and carbohydrates. Each formulation contains a mineral and vitamin mix to improve micronutrient intake, the milk minerals provided by the whey permeate in two of the supplements are in addition to the standard amount provided. In as much as is possible, the supplements are designed to have a similar appearance, texture, colour and taste. Table 1 provides an overview of the LNS interventional product compositions.

## Rationale for use of LNS-LQ

LNS is a nutrient dense fortified lipid-based paste that is produced to meet high quality and safety standards. It is well adapted for use in homes where few resources are available, for example; LNS has a long shelf life, is packaged in daily portion sizes, does not require refrigeration, does not require preparation, mixing or diluting and can be consumed directly from the sachet.

## Dosage

Children randomised to LNS will be allocated one 100g sachet of LNS-LQ (530-535kcal) per day for 12 weeks.

## Rationale for dosage

The energy need per kg body weight for healthy 1-5 year-old children is around 100 kcal/kg/day with slightly higher needs at 1 year decreasing with age (<http://www.fao.org/3/aa040e/AA040E07.htm>). To provide enough nutrients for catch-up growth, the formulation has been designed/selected to meet up to 50% of the total energy requirements and 100% of micronutrients needs [26]. In addition, the dose of milk protein and whey permeate in the LNS-LQ corresponds to approximately 20-25 g milk powder or 1 glass of milk per day, which has been associated with increased linear growth in other studies [27]. We do not foresee a risk of overweight. In one of our previous studies, short children with low MUAC aged 6 – 23 months gained mainly fat-free mass and not excessive fat mass after intake of a similar dose of energy and nutrients [28]For treatment of children aged 12-59 months with MAM, it is recommended to provide 75 kcal/kg body weight, approximately half of the daily energy requirements (to supplement the family diet) and enough micronutrients to cover all of the child’s daily requirements . Children with MAM measuring between 65 and 87cm can weigh between 5.5 and 10.4 kg , and therefore require a daily supplement of between 413 kcal (5.5 kg * 75 kcal/kg) and 780 kcal (10.4 kg * 75 kcal/kg). Our dose of 540kcal is in line with the current WFP (510-560kcal/100g) and USDA (520-550kcal/100g) recommendations for daily supplementation of MAM.

## Administration of study treatment

At inclusion, clear instruction is given to the caregiver on how to safely and correctly give the supplement to the participating child in the home. These messages are reinforced throughout the duration of supplementation. The SOP describes the messages being communicated.

## LNS-LQ nutrition composition

| **Table 1**: The nutritional composition of the four lipid-based nutrient supplements (LNS-LQ) | | | | | |
| --- | --- | --- | --- | --- | --- |
|  | Name | **Milk protein and whey permeate**  **LNS-LQ** | **Milk protein and no whey permeate**  **LNS-LQ** | **Soy protein and whey permeate LNS-LQ** | **Soy protein and no whey permeate**  **LNS-LQ** |
| Macronutrients | | | | | |
| Components | Unit | Per 100g | | | |
| Calories | Kcal | 531 | 535 | 530 | 534 |
| Carbohydrates | g | 42 | 43 | 42 | 43 |
| Lactose | g | 15.7 | 0.4 | 15.3 | 0 |
| Proteins | g | 13.9 | 13.5 | 13.9 | 13.5 |
| Milk proteins | g | 7.15 | 6.75 | 0.40 | 0 |
| Vegetable proteins | g | 6.75 | 6.75 | 13.50 | 13.50 |
| Lipids | g | 33.7 | | | |
| Linoleic acid C18:2 | g | 3.0 | | | |
| Alpha-linolenic acid C18:3 | g | 0.5 | | | |
| Minerals | | | | | |
| Calcium | mg | 691 | 594 | 691 | 594 |
| Copper | mg | 1.65 | | | |
| Iron | mg | 12 | | | |
| Iodine | µg | 127 | 113 | 127 | 113 |
| Magnesium | mg | 199.2 | 175.8 | 199.2 | 175.8 |
| Manganese | mg | 1.8 | | | |
| Phosphorus | mg | 661 | 539 | 661 | 539 |
| Potassium | mg | 1315 | 985 | 1315 | 985 |
| Sodium | mg | 84 | 7 | 156 | 79 |
| Selenium | µg | 30 | | | |
| Zinc | mg | 12.5 | | | |
| Vitamins | | | | | |
| Vitamin A | mg | 619 | | | |
| Vitamin B1 | mg | 1.2 | 1.1 | 1.2 | 1.1 |
| Vitamin B12 | µg | 3.2 | 3.0 | 3.2 | 3.0 |
| Vitamin B2 | mg | 3.1 | 2.8 | 2.7 | 2.4 |
| Niacin | mg | 14.9 | 14.6 | 14.9 | 14.6 |
| Pantothenic acid | mg | 5.7 | 4.5 | 5.7 | 4.5 |
| Vitamin B6 | mg | 2.1 | 2.0 | 2.1 | 2.0 |
| Biotin | µg | 74.1 | 67.6 | 74.1 | 67.6 |
| Folic acid | µg | 223 | | | |
| Vitamin C | mg | 67.9 | 67.6 | 67.9 | 67.6 |
| Vitamin D | µg | 16.9 | | | |
| Vitamin E | mg | 18 | | | |
| Vitamin K | µg | 30 | | | |

# Reference group

This study includes a group of 150 un-supplemented stunted children as a reference group. These children will not receive an intervention product, but instead will continue with the family diet and will receive locally adapted nutrition counselling as well as monitoring and regular follow-up over the intervention period.

# Deterioration and referral

Children identified as having SAM (MUAC < 115 mm OR WHZ <-3 OR bipedal pitting oedema) prior to inclusion, or participants in the study who deteriorate to SAM (without other complications) are referred for outpatient treatment of SAM (OTC). If the child also has complications, they are referred to inpatient care (ITC). Those identified as having MAM but who are younger than the recruitment age, will be referred to local NGO services or other health facilities for caregiver nutritional/social support (where available). All participants requiring hospital attention will be referred for treatment and attempts will still be made to follow-up on the child wherever possible.

# Concomitant care for study participants

Participants will not be provided with any medical treatment by the study. Wherever treatment is needed, children are referred for the necessary medical attention.

# Strategies to improve and monitor adherence

It is a challenge to ensure that the participating child is the one receiving the LNS, due to home based caregiver administration. As such, several actions are taken to improve compliance.

- Siblings: We aim to only include one child per household in the study. However, if there are other young children in the household (6 months to five years), instructions and sachets will be given so that the caregiver receives extra rationing. This strategy aims to minimize the likelihood of sharing.
- Identification: Several methods are used to ensure that the correct child is matched to the correct study records, namely wrist bands and photo id. The SOP describes the use of these processes in detail.
- Accountability: Any distribution of sachet stocks will be logged by study staff. Additionally, when collecting new sachets, the caregiver is requested to return the empty sachets from their previous two week supply. This is to improve accountability on the part off the caregivers, and encourage against sachet redistribution or selling.
- Monitoring adherence: Trained study staff will make regular phone follow-up calls to the participant/caretakers to remind them of their clinic visits and enquire about stool sample collection or LNS adherence where applicable. At clinic visits and when the caregiver is collecting sachet stocks study staff will also enquire about LNS adherence.
- Incentives: Throughout the study, the child’s health will be monitored regularly. A travel reimbursement of not more than 20,000UGX will be provided to cover the cost of return transport and includes some allowance for e.g. food purchase during the day while at the clinic visit. Any caregivers attending with unsupplemented children will also receive a small token which can be appreciated by the whole family, for example clothes soap. The value will not exceed 10,000UGX.

# Outcomes

All outcomes are measured over time from baseline to 12 weeks.

## Primary outcome

- Knee-heel length (mm)
- Length/height velocity(cm)

## Secondary outcomes

- MUAC (mm)
- Height-for-age z-scores (HAZ)
- Weight-for-height z-scores (WHZ)
- Weight-for-age z-scores (WAZ)
- Child development index (CDI)
- Head circumference over time
- Weight gain (g)
- Haemoglobin (Hb)
- Body composition
- Bioimpedance: FM (kg), FFM (kg), FMI (kg/m^2^), FFMI (kg/m^2^)
- Skin folds: triceps, subscapularis mm/week

## Tertiary outcomes

Blood hormone markers

- Insulin-like Growth Factor-1 (IGF-1)
- Insulin

Blood markers of systemic inflammation

- C-reactive protein (CRP)
- Alpha-1-acid glycoprotein (AGP)

Blood markers of enteric function

- Citrulline.
- Blood markers of micronutrients status Iron (ferritin and soluble transferrin receptor)
- Vitamin B12
- Folate
- Vitamin A (retinol binding protein)
- Stool markers of intestinal inflammation Myeloperoxidase (MPO)
- Neopterin (NEO)
- Alpha-1-antitrypsin (AAT)

Gut microbiota

Safety, morbidity and loss to follow-up

- Proportion deteriorated to MAM, measured as WHZ ≤ -2 and >-3 or MUAC ≤ 125 and >115mm) and proportion deteriorated to SAM, measured as WHZ <-3, MUAC <115mm or presence of nutritional oedema.
- Proportion of participants who die during the study period (mortality)
- Number of morbidity episodes, duration and severity (diarrhea, pneumonia, fever, malaria, other) during the intervention period
- Proportion lost to follow-up during the study period

## Other assessments

Additional information collected at baseline:

- Demographics, medical history
- Physical examination
- Dietary intake assessment
- WASH assessment taken at initial home visit
- Intermittent follow-up on child health status and compliance throughout study

# Sample size

The sample size calculation is generic, based on the ability to detect differences in terms of standard deviations. Conservatively, we want to be able to detect a 0.35 SD or greater difference between any two groups with 5% significance and 80% power. For this, we need 129 children in each group, but to allow for 10% loss to follow-up, we will include 150 children in each group. However, given the factorial design, if there are no interactions between the two experimental interventions, we will be able to compare two groups of 300 children each, which will enable us to detect a difference as small as 0.24 SD. In the Treatfood trial, the SD of knee‐heel length at baseline was 18.1 mm [17]. Hence, a 0.24 SD difference corresponds to 4.3 mm. In secondary analysis, we will compare the 600 supplemented children with 150 unsupplemented. These group sizes will allow us to detect a 0.27 SD difference, corresponding to 4.9 mm.

# Schedule of measurements and assessment

**
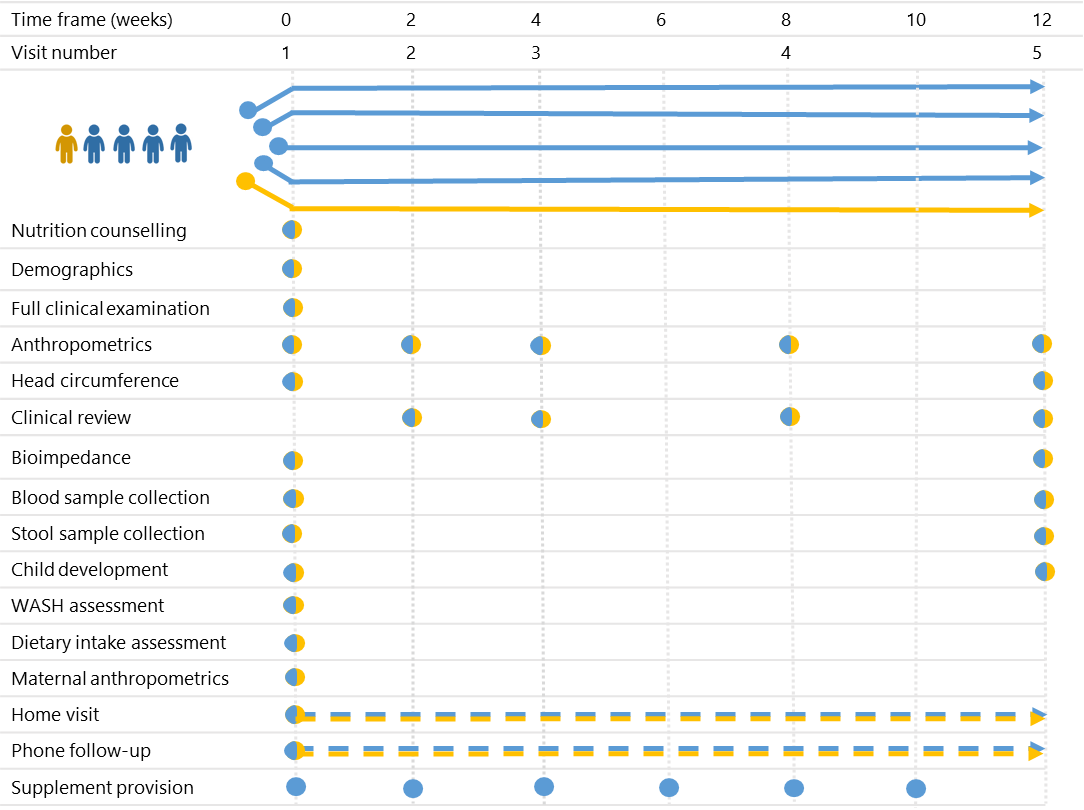
**

**
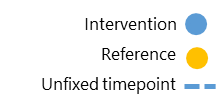
Figure 4: MAGMAM data collection time-points and visits.**

Timeline for enrolment, interventions, assessments and visits for each participant enrolled in the study. Phone follow-up and home visits are unfixed time points depending on need and availability. Time points are considered valid if taken within ±7 days of baseline, week 2 and week 4 and ±14 days from all other time points.

# Assignment of interventions

## Stratified block randomisation

Site-stratified, block-randomisation will be used to allocate children to the four intervention groups and the reference group. Each site will have a separate randomization list. This ensures that the numbers receiving each supplement or no supplementation will be closely balanced between groups and at each of the study sites. In order to maintain the unpredictability of the random sequences, large and randomly varied block sizes will be used.

## Allocation sequence generation

The allocation sequence will be computer generated using [www.randomization.com](http://www.randomization.com) or a similarly appropriate computer randomisation system, prior to study start. The sequence will be generated by a member of staff at the University of Copenhagen in Denmark, who is external to implementation of the study.

## Allocation sequence concealment

The allocation sequence is not accessible to study personnel besides the product distribution staff. They are able to see the product code for treatment assignment but are unable to make any changes to allocation and will not have access to the blinding code. A member of staff at the University of Copenhagen, who is external to data collection, will keep a secure copy of the allocation sequence.

## Blinding

This is a double blinded study. Both the study staff and the caregivers are blinded to which intervention is allocated to the study participant. Products are designed to be as indistinguishable as possible. The reference group is aware that they are not allocated to an intervention, however, the clinical study staff will not be made aware of which group participants are allocated to.

## Product codes

The four LNS-LQ products are each allocated two different product codes so that there are 8 product codes in total. The product code document, stating which product corresponds to which product codes, is prepared and placed in sealed envelopes by Nutriset. These product codes are kept locked and will first be revealed following the primary analysis.

## Product distribution

Product distribution takes place away from other study operations. The product distributor/s are solely responsible for product distribution and product monitoring at each site. They will not be involved in any clinic related data collection. In the interest of compliance, caregivers are requested to return empty sachets to the product distributor at each visit. The distributor keeps a confidential supplement distribution log.

# Pilot phase

A pilot will be conducted prior to the study start. This is done in order to test the study procedures, CRFs and SOPs are operational and clear to all who will use them. During the pilot phase of the study, a maximum of 20 children will be enrolled. Study procedures will be carried out in order to test the protocol, questionnaires and staff roles. Importantly, there are two exceptions to following the protocol during the pilot: 1. No blood samples will be collected during the pilot phase and 2. Intervention products will not be used. Data collected during this period will be stored separately and will not be used as part of the study data.

# Data collection and measurements

## **A**ge and birth weight

In order to gather information on the child’s exact age and birth weight, caregivers are requested to provide a birth information card for that child. If this is not available, study staff will attempt to identify the child’s age through a series of questions.

## Sociodemographic and medical questionnaire

At baseline an interviewer-administered questionnaire is used to collect information on participant socio- demographic characteristics and medical history. A medical history questionnaire is also to be administered at 12 and 24 weeks.

## Clinical examination

A thorough physical examination is carried out at baseline including rapid tests for HIV and Malaria and assessment for vital signs (pulse, blood pressure and respiratory rate). At follow-up clinic visits thereafter, a short clinical review is conducted including information on recent medical history and milk consumption. If an illness is suspected, a treatment is recommended but not provided by the study. If necessary, the child is referred for treatment. Where relevant, the resolution/worsening of adverse events is monitored by clinic staff. The SOPs describe the process in further detail.

## Anthropometrics

Anthropometrics will be taken in repeated measures and on the left side of the body wherever possible. To reduce variation in the measurements, in as much as is possible, the same study staff will take anthropometric measurements, each having been specifically trained in anthropometrics. All equipment is calibrated regularly and the same equipment used (wherever possible) between study sites. All anthropometric measurements are feasible in the study context and are not invasive or harmful to participants.

### Child anthropometry

The following anthropometrics are taken at baseline, week 2, 4, 8 and 12 weeks; knee-heel length, MUAC, weight, height, skin folds (subscapular and triceps). The distance between the heel and knee is measured using a digital hand-held knemometer (Michaelsen KM et al, 1991). Knee-heel length is a sensitive measure of change in growth over short time periods, detecting smaller differences with less variation than overall height. Weight is measured using an electronic scale with a double weighing function. Height (measured in children 24 months and older) and length (measured in children <24 months) are taken using a standard wooden height/length board. The anthropometric indices HAZ, WAZ and WHZ are used to monitor nutritional status over time according to the WHO growth standards. Mid-upper-arm circumference is measured using a MUAC tape. A low MUAC has been associated with higher mortality risk in young children [29]. The MUAC has a high specificity and gives an indication of wasting and of lean mass accretion over time. Skin fold thickness is measured in the triceps and subscapular using a Harpenden skinfold caliper. Head circumference is measured using a non-stretch measuring tape at baseline, week 12 and week 24. Head circumference can be useful when interpreting child development outcomes.

### Maternal anthropometry

If the mother is available, her weight and height will be measured at baseline (or when available) using electronic scales and an adult height board according to the SOP. Maternal anthropometrics have been associated with the growth of their offspring and it is therefore of interest to measure maternal BMI in this study [30].

## House mapping and WASH assessment

At the first participant home visit study staff record the GPS coordinates of the home site location, in case home follow-up is needed at a later stage. Study staff also make a short assessment of WASH characteristics including; water source, basic sanitation and use of soap. More information is provided in the SOP.

## Nutrition assessment

General nutrition intake is assessed at baseline using an adapted food frequency questionnaire. The food frequency questionnaire gives an indication of breastfeeding status and diet.

## Bioimpedance

Fat mass and fat-free mass are measured using bioimpedance, at baseline and 12 weeks. Bioimpedance is taken while the child is lying down, preferably after 10 minutes rest. Once the reading begins, it takes less than 10 seconds to complete. A maximum of three repeated measurements are taken and all measurements are carried out by trained staff in accordance with the SOP. Electrode stickers are placed at the dorsal surface of the hand and foot. Impedance is measured through the right side of the body where possible (stickers placed on the right side). The current passed is not harmful and cannot be felt. The equipment is approved for medical use according to European standards.

## Child development

The Malawi Developmental Assessment Tool (MDAT) is a culturally relevant, reliable and sensitive child developmental assessment tool for use in African settings (Gladstone et al., 2010). The MDAT has been adapted and validated for use in Uganda. Child development index (CDI) is calculated at baseline and 12 weeks using a version of the MDAT that is both in English and translated to Lusoga. A study staff member trained in child development assessment will assess according to the SOP. The tool focuses on four domains; gross motor, fine motor, language and social development. An overall score for each domain is given according to the number of items passed.

## Phone call follow-up

When appointments are made, either at recruitment, inclusion or follow-up, caregivers are asked for phone contact information in order to follow-up on the appointment attendance. If included, selected study staff will attempt to establish phone contact to enquire about recent medical history, milk consumption and to monitor stool sample collection, LNS-LQ compliance or the resolution/worsening of adverse events where applicable.

## Sample collection and storage

### Stool

Stool samples are collected for later analysis of markers of gut function and microbiota, see table 2 for an overview of stool sample collection. Approximately 5-10g is collected from the study participant at two time-points; at baseline and at 12 weeks. Caregivers are given a sample collection kit and instruction on how and when to collect and return samples. Samples may be collected during the respective clinic visit if available. The sample may be stored at room temperature for up to 5 days after collection. Further details concerning sample collection are provided in the SOP. Stool samples are stored at −20 °C until delivery to the main storage site in Kampala where samples are then stored at −80 °C until they can be shipped internationally for analysis. See figure 5 for an overview of stool sample cold chain process.

**
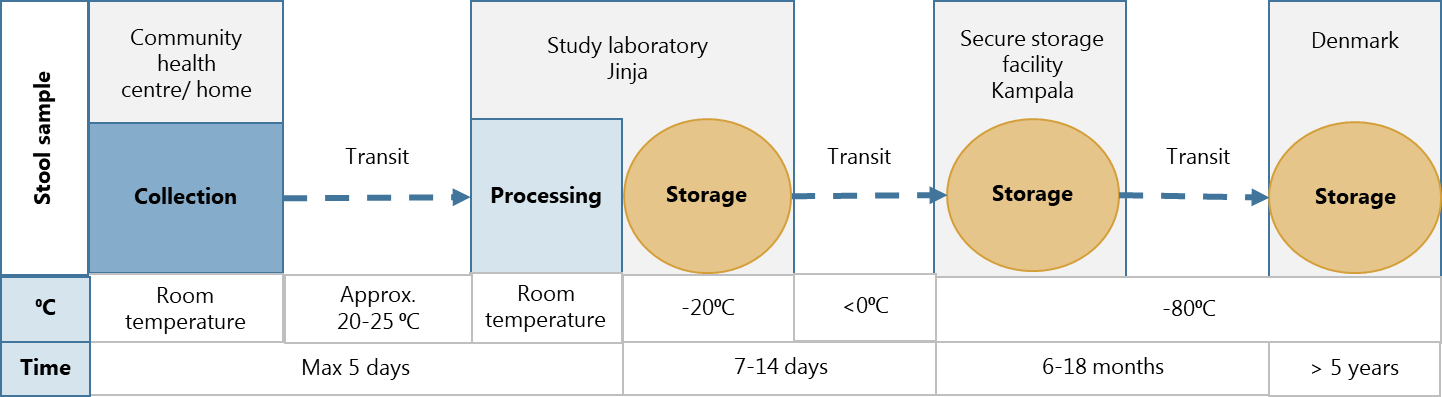
**

**Figure 5**: Stool sample flow, including approximate time frames and approximate temperatures

### Blood

Blood samples are collected and stored for later measurement of selected growth factors, nutrient status, markers of gut function and markers of inflammation (table 2). Samples are collected by trained study nurses at baseline and at 12 weeks. A total of 6.0 ml of venous blood is collected by standard phlebotomy.

A drop of blood will be used to measure HIV status (preferably at baseline). Caregivers will be referred to the government health clinic for HIV counselling services where applicable. Whole blood haemoglobin status will be measured using a Hemocue at baseline and at 12 weeks. Additionally, all participating children will be tested for malaria at baseline using a RDT test. Further details concerning sample collection are provided in the SOP.


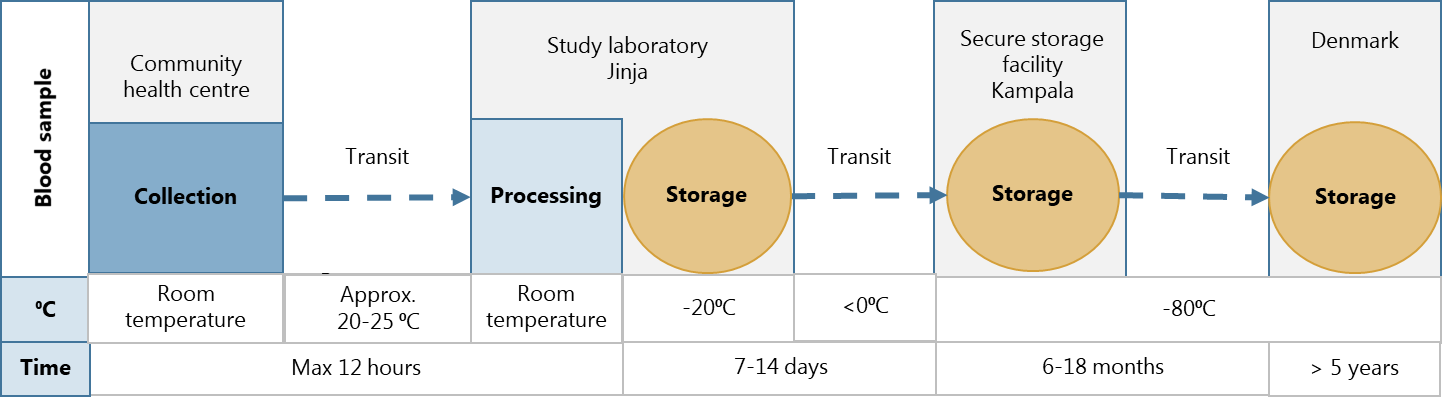


**Figure 6**: Blood sample flow, including approximate time frames and approximate temperatures

### Transportation and shipment of samples

Once a week or as frequently as possible, blood and stool samples are transported from the laboratory in Jinja to a secure storage facility in Kampala where they are stored at -80⁰C until they can be shipped internationally for later analysis. Prior to shipment of samples, a materials transfer agreement (MTA) will be established. Samples are collected in storage and shipped in larger shipments. All samples are kept at -80°C until shipment, and are stored at -80°C while they are awaiting analysis in Denmark. Samples are stored until minimum 5 years after study termination.

### Sample collection and analysis plan

| **Table 2: Sample collection and analysis plan** | | | | | |
| --- | --- | --- | --- | --- | --- |
| Collection | | | | Analysis | |
| Sample  Collected | Test | When to collect | Type of sample | Suggested method of analysis | Site of analysis |
| Blood sample, 6 ml | HIV status | Baseline | Whole blood, drop | RDT Antibody (all ages) + PCR (only <18 months (if available)) | Uganda – clinic |
|  | Malaria | Baseline | Whole blood, drop | RDT – smear |  |
|  | Hb, | Baseline and  week 12 | Whole blood, drop | Hemocue RDT |  |
|  | IGF-1, insulin |  | Serum/plasma | ELISA | Shipment abroad after study completion |
|  | CRP, AGP |  | Serum | ELISA |  |
|  | Iron status (SF, sTfR), vit A |  | Serum | ELISA |  |
|  | Citrulline |  | Plasma/serum | LC/MS/MS |  |
|  | Folate and vitamin B-12 |  | Plasma | ELISA |  |
|  |  |  |  | ELISA |  |
| Stool sample, approx.1-5 g | MPO | Baseline and  week 12 | Fecal sample supernatant, 1.0g | ELISA | Shipment abroad after study completion |
|  | NEO |  | Fecal sample supernatant , 1.0g | ELISA |  |
|  | AAT |  | Fecal sample supernatant , 1.0g | ELISA |  |
|  | Gut microbiota |  | Fecal sample supernantant , 1.0g | HTS-DNA PCR |  |

RDT: Rapid diagnostic test; PCR: Polymerase chain reaction; ELISA: Enzyme-linked immunosorbent assay; LC/MS/MS: Liquid chromatography tandom mass spectrometry; Hb: Haemoglobin;; IGF-1: Insulin Growth Factor-1; CRP: C-reactive protein; AGP: Alpha-1-acid-glycoprotein; SF: Serum ferritin; sTfR: Soluble transferrin receptors; MPO: Myeloperoxidase, NEO: Neopterin; AAT: Alpha-1-antitrypsin; HTS-DNA: High throughput DNA sequencing

## Deterioration and loss to follow-up

*Proportion deteriorated to MAM or SAM* is the proportion of children who have deteriorated according to their status at inclusion. Either to MAM, measured as MUAC < 125 mm and ≥115mm or WHZ between -3 and -2, OR the proportion of children who have deteriorated to SAM, measured as WHZ <-3, MUAC <115mm or the presence of nutritional oedema.

*Proportion and qualification of morbidity* is the number of episodes, number of days with and the qualification of morbidity, during the 12 week intervention period. During the baseline clinical examination, signs and symptoms of the following are assessed: pneumonia (moderate or severe), diarrhea [acute watery (<14 days), acute bloody (<14 days), persistent (≥14 days)], fever (>38⁰C), common cold and other. The caregiver is given an exercise book at inclusion to be used to record morbidity information if the child visits another clinic or is admitted. If the caregiver brings this book to the clinic visit, it can be used to inform the clinician. In addition, caregiver perceived morbidity is assessed (when possible) with phone call follow-up over the 12 week intervention period.

*Mortality* is the proportion of participant deaths recorded during the 12 week intervention period.

# Quality assurance

Study personnel will be trained in study procedures before they can participate in data collection or study conduct. The following list provides an overview of some of the procedures described in SOPs included in the training. Note that this is not an exhaustive list.

- Recruitment and referral
- Screening and inclusion
- Randomization and blinding
- Nutrition counselling
- Training caregivers in the correct storage and use of LNS & LNS distribution
- Training caregivers on stool collection at home
- Anthropometry
- Bioimpedance
- Sample (blood and stool) collection, labelling and storage
- Child development
- Clinical follow-up
- Phone follow-up
- WASH assessment
- Adverse event registration
- Discharge
- Data entry and data management
- LNS storage and disposal

# Data management

## Data entry

All participant data are collected on a paper case report form (CRF) and are double entered using Epidata software (<https://www.epidata.dk/>). The secure electronic data collection platform REDCap (Open Source Vanderbelt University) may be used for specific tasks such as participant registration and randomisation. However, these processes can be carried out without REDCap should an internet connection be unavailable. Data should be primarily entered into the CRF and not written down elsewhere unless stipulated in the SOP.. All study staff are trained extensively in how to fill in the sections of the CRF that are relevant to their role. Selected senior staff members are responsible for ensuring that data entry is complete.

## Coding

Data integrity is enforced with inbuilt value checks, data ranges and consistency checks in Epidata..

## Data security

If data is changed on a paper CRF, it is signed by the respective staff member in case a query arises. After a participant has been discharged from the study, both the informed consent form and hard copy of personal identifiers are kept in a secure and safe location away from the rest of the data. Hard copies of the CRFs are source data and are to be stored in numerical order according to the ID number in a secure but accessible place. All source data is kept securely on file for a minimum period of 5 years after completion of the study. The REDCap system is centralised at the University of Copenhagen on secure online servers which are monitored and backed up regularly.

## Data safety monitoring board

An independent data safety monitoring board (DSMB) has been established and all members have consented to participation on the board. The DSMB consists of four members:

- Chairman: Dr James Bunn, Paediatrician, World Health Organisation, Sierra Leone.
- Board Member: Dr Marko Kerac, PhD, Assistant Professor and MSc Nutrition for Global Health Programme Director, London School of Hygiene & Tropical Medicine, London, UK.
- Board Member: Dr. Erisa Mwaka, PhD, bioethicist, senior lecturer at the College of Health Sciences and Chair of Biomedical Sciences Higher Degrees and Research Ethics Committee, Makerere University.
- Board Member: Charles Opondo, BPharm, MSc and PhD in Medical Statistics. Honorary Assistant Professor at the Department of Medical Statistics, London School of Hygiene & Tropical Medicine, London, UK

The DSMB is a group independent of the study organisers who will monitor safety parameters. They will receive unblinded data including individual case fatality reports, SAEs and reasons for termination of participants. Based on this information, the DSMB will decide if the study can continue or if any actions are needed. There is one pre-defined stopping rule for the unlikely risk of high mortality: p< 0.01 after inclusion of 300 children. The safety monitoring board will have access to the randomization code and will not be blinded to the treatment of the children.

# Statistical methods

Primary, secondary, and tertiary endpoints are analysed by means of longitudinal data analysis accounting for the fact that repeated measurements are acquired per participant. Missing data will be handled by intention to treat analysis. A statistical analysis plan will be prepared before unblinding of the trial.

# Assessing and reporting adverse events and reactions

Serious adverse events (SAEs) and adverse events (AEs) are collected in accordance with what is outlined below, as per the guidelines from SOMREC and in accordance with the SOP.

## Adverse events

In this trial, an adverse event constitutes any untoward medical occurrence in the clinical trial subject at any dose of the LNS nutritional intervention product and which does not necessarily have a causal relationship with this treatment. The AEs will be recorded in the CRF.

- All reported or observed adverse events (AEs) taking place during the intervention period of the trial will be recorded; this is from the time informed consent is given, until the time when the participant is discharged from the study.
- The PI will report all AE records to the sponsor biannually. The sponsor will keep detailed records of adverse events.
- AEs will be reported to SOMREC in the annual progress reports.

## Serious adverse events:

A serious adverse event (SAE) in this study is any untoward medical occurrence that at any dose of the LNS nutritional intervention: results in death, is life-threatening (immediate risk of death), results in persistent or significant disability or requires inpatient hospitalisation or prolonged hospitalisation.

- SAEs occurring to a study participant during the trial period will be reported to the sponsor within two working days of becoming aware.
- SAEs will be reported to SOMREC within 7 calendar days of becoming aware of the event. If more information concerning the event becomes available, a follow-up report will be submitted to SOMREC within 14 calendar days of becoming aware of the event.
- Serious adverse events occurring after a subject is discontinued from the study will NOT be reported unless the investigator suspects that the event is related to the study investigational products.

## Suspected unexpected serious adverse reactions:

A suspected unexpected serious adverse reaction (SUSAR) is a suspected untoward and unintended response to an investigational medicinal product related to any dose administered.

- All SUSARs are reported to the sponsor within 48 hours
- Within 7 calendar days of becoming aware of the event, the SUSARs will be reported to and to SOMREC.
- If more information concerning the event becomes available, a follow-up report will be submitted to SOMREC within 14 calendar days of becoming aware of the event.

# Monitoring

The study intends to have internal on site monitoring at initiation, during and after termination of the study. Monitoring is carried out according to the monitoring plan. Remote monitoring will be conducted from Denmark.

# Ethics and dissemination

## Assessment of anticipated benefits and risks

Clinical benefits: It is anticipated that all children participating in this study will benefit from having close monitoring from doctors and study staff.

Community benefits: It is anticipated that the training of VHTs and clinical staff within the community will build capacity and enhance their awareness of the monitoring for child malnutrition, encouraging the practice at community clinic sites in the future.

Study risks: Deterioration to SAM. There is a very low-risk for deterioration to SAM. Such deterioration would likely be associated with another factor such as HIV or severe acute illness which are likely to be few in this population group.

Product risks: The intervention products are not expected to pose any risk to study participants. The only possible risk scenario would be if the child had an unknown allergy to an ingredient. This however is unlikely since serious allergies to peanuts or milk would likely already have been discovered prior to joining the study. There is a very low-risk for excessive weight gain (>2 WHZ) when assigned to an LNS-LQ product. If this occurs, we expect it to be in few cases who may take on weight first before linear growth is achieved. We do not expect this to have any negative long term consequences.

Procedural risks: There are very few procedural risks in this study. Blood samples are collected by trained nurses at two time points (at 0 and 12 weeks). Some temporary discomfort is experienced by the child when blood samples are taken but no other risk is expected.

## Discontinuation of participation in the study

Parents or caregivers are allowed to withdraw their child from the investigation at any time for whatever reason. The Investigator also has the right to discontinue participant investigations at any time if they deem that it is in the best interest of the participant or if non-compliance with the clinical protocol impacts on the scientific integrity of the clinical study. Possible reasons for premature withdrawal of a study participant include:

- Non-compliance with the clinical protocol impacting on the scientific integrity of the clinical study.
- If a child’s safety and well-being is compromised by further participation

In case of patient withdrawal, the CRF will be completed up to the point of patient withdrawal and all available data will be used in data evaluation/statistics. Withdrawn patients will not be replaced.

All participants requiring hospital attention will be referred for treatment. If a child is referred for hospital treatment, attempts will still be made to follow-up on the child wherever possible.

Early termination of a child from the study occurs due to the following reasons: the investigator sees fit to discontinue participation, the caregiver wishes to discharge, death and loss to follow-up.

If a child dies while enrolled in the study, the medical cause will be identified (if possible) by verbal autopsy, noted in the CRF and reported to the sponsor (as per the SAE reporting SOP).

## Research ethics approval

The study is conducted in accordance with the ethical principles set forth in the current version of the Declaration of Helsinki and all applicable local regulatory requirements. The rights, safety and well-being of the children involved in the study will prevail over science and society.

The study has been submitted for approval to the School of Medicine Research Ethics Committee at Makerere University and The Ugandan National Council of Science and Technology (UNCST). The study has also been submitted for consultative approval from the Danish National Committee on Biomedical Research Ethics. The study will only be initiated after all authorities concerned have approved the study.

This clinical study follows Ugandan laws of data protection. All sensitive information collected during the course of this study is to be kept strictly confidential. Study participants are identified by participant ID number and any identifying information is archived with study documents. Each participant remains anonymous during data analysis.

Should the study require review, relevant regulatory authorities and ethics committees are allowed to access all relevant information for audit and inspection purposes.

## Training in good clinical practice (GCP)

All study staff who are directly involved in the collection of participant data will undertake a course in GCP. The principle investigator and co-investigators directly involved in participant interaction will be trained in Human Subject Protection (HSP) prior to study start. Other staff, directly involved in participant care, will receive HSP training within 1 month of joining the study.

## Protocol amendments

Substantial amendments to this protocol can only be made after the IRB have given approval for the changes. Amendments to the protocol are regarded as substantial if they have significant impact on:

- The safety, physical health and mental integrity of the study participants
- The scientific value of the study
- The conduct or the management of the study
- The quality or safety of the investigational product (IP) used in the study

Any amendments to this protocol will be signed by the signatories included in section 2. If any event occurs related to the conduct of the study or the development of the test product which may affect the safety of the children, the investigator may take appropriate measure to protect the participants against immediate hazards without notifying the IRB first. The IRB will be informed as soon as possible thereafter.

## Protocol deviations and violations

Protocol deviations are unplanned instances of non-compliance to the protocol. These are noted during the study, recorded and evaluated as major or minor before the blind of the study is broken. Any major protocol violations are reported to SOMREC within seven (7) calendar days of becoming aware of the event (in accordance with UNCST guidelines). All minor deviations from SOPs or the protocol will be reported in “Notes to file” and submitted with the annual progress reports to SOMREC, e.g. if a participating child does not provide a stool sample on time. Participants with major protocol deviations or violations are excluded from Per-Protocol-statistical analysis.

## Participant informed consent

Written, informed consent is obtained from all study participants prior to entry into the study. A parent/caregiver will consent on behalf of the participant, according to Ugandan laws and guidelines. Emancipated minors (mothers under 18 years old) can also consent on behalf of the child in their care. Participants are screened for eligibility to the study and then approached for possible participation in the study. See section 7.2.2 for more detail. Before a child can be included, the delegated study personnel will explain verbally to parents/caregivers, in relevant local language; the objectives, nature, risks and implications of the study. The same information will be described in the study participant information sheet. All information material will be translated to Lusoga. To ensure that caregivers understand the information provided during the informed consent process, they will be asked a short follow-up questionnaire by a study staff member who did not carry out the informed consent process with the caregiver.

In particular, the participants will be informed about the following:

- The possibility of withdrawing from the study at any time
- How the sensitive and health-related data will be collected and used during the study
- Follow-up procedures

The participants are given time to discuss any concerns or questions they may have and make a decision regarding the participation of the child in their care. If the parents/caregivers are illiterate, a fingerprint will be accepted instead of a signature. In this case, a literate witness is required to be present during the reading of the consent form and to sign the consent form in addition to the caregivers fingerprint.

All parents/caregivers receive a copy of the participant information sheet and the signed consent form. The original is retained by the investigator. The informed consent form includes contact information of the study PI should the caregiver have further questions.

Information about the participants is kept confidential and not disclosed to anyone outside the study team and clinical staff, unless explicit permission is obtained from the caretaker. When study assessments identify an abnormal condition requiring treatment, relevant data may be provided to clinical staff so that actions may be taken to treat the condition.

## Photos and video permissions

On the day of inclusion, caregivers (who have given consent for study inclusion) will be asked if they give permission for photos and videos to be taken of them and their participating child for use as a complement to research aims. To be shared in situations such as conference or publication settings. The caregiver may also consent for a photo to be taken of them and their child for purposes of identification during the study. All photo permissions are completely voluntary and do not impact on whether a participant will be included or not. The caregiver can opt to withdraw consent for photos/videos at any time.

## Permissions to contact for later follow-up

On the day of inclusion, caregivers will be asked if they give permission for the study team to contact them again after study completion for one or more follow-up study/studies. The follow-up studies may take place after 1-2 years and later. Permissions are completely voluntary and do not impact on whether a participant will be included or not. The caregiver can opt to withdraw consent for us to contact them at any time.

## Confidentiality and data handling

All study-related information is stored securely; either in a database or on site with secure access. To maintain participant confidentiality, all laboratory specimens, reports, data collected and administrative forms are identified by an ID code only. All records that contain names or other personal identifiers, such as locator forms and informed consent forms, are stored separately and securely from study records and remain in Uganda. All local databases are secured with password-protected access systems.

The study includes researchers from Uganda and Denmark. Results of the assessments and results of analysis of blood and stool samples will be shared between the researchers. Data handling in Uganda is performed under the Ugandan Data Protection and Privacy Act. When data and samples are handled in Denmark, researchers work under the EU General Data Protection Regulation and data are processed according to Article 9, (2), (a) of the General Data Protection Regulation. When data are returned to Uganda, data handling is no longer within the scope of EU regulation.

## Disposal of the LNS-LQ

As this is a food product, if the supplement has a minimum shelf-life remaining, it may be donated. Expired nutritional product will be disposed of.

## Declaration of interests

The investigators declare that they do not have any financial interest in the results of the study and no affiliation with or financial interests in dairy industries or organisations, including Arla Foods.

## Compensation/Reimbursement

Participation in the study is free of charge and caregivers do not receive any payments for participation in the study. Caregivers will be reimbursed for their transportation costs which will include a small amount to cover the costs of food purchase during the day of the clinic visit. A maximum of 20,000UGX will be given per return trip. Caregivers who do not receive LNS-LQ supplementation for their child during the study will still receive the same opportunities for medical review. In addition, a small token of appreciation, worth not more than 10,000 UGX will be provided to families of unsupplemented children.

## Insurance

Insurance has been agreed upon with a local Insurance company in consideration of the nature of this trial.

## Dissemination policy

After completion of the study, the results will be published in abstracts, posters, magazines, web pages or scientific articles irrespective of positive, negative or inconclusive data. In all cases participant identity will remain confidential. Publication of scientific articles will follow the Vancouver rules of publication. Data from the investigation is considered confidential until it is published.

# References

1. UNICEF, WHO, World Bank. Levels and trends in child malnutrition: key findings of the 2019 Edition of the Joint Child Malnutrition Estimates. Geneva: World Health Organization; 2019 Mar p. 16.

2. WHO Multicentre Growth Reference Study Group. WHO Child Growth Standards: Methods and Development. Geneva: World Health Organization; 2006 p. 312. Available: http://www.who.int/childgrowth/standards/Technical_report.pdf?ua=1

3. McDonald CM, Olofin I, Flaxman S, Fawzi WW, Spiegelman D, Caulfield LE, et al. The effect of multiple anthropometric deficits on child mortality: meta-analysis of individual data in 10 prospective studies from developing countries. Am J Clin Nutr. 2013;97: 896–901. doi:10.3945/ajcn.112.047639

4. Humphrey JH, Prendergast AJ. Population-level linear growth faltering in low-income and middle-income countries. Lancet Glob Health. 2017;5: e1168–e1169. doi:10.1016/S2214-109X(17)30425-4

5. Uganda Bureau of Statistics. Uganda Demographic Health Survey, 2016. Key Indicators Report. Kampala, Uganda and Rockville, Maryland, USA: UBOS and ICF; 2016 p. 72.

6. Danaei G, Andrews KG, Sudfeld CR, Fink G, McCoy DC, Peet E, et al. Risk Factors for Childhood Stunting in 137 Developing Countries: A Comparative Risk Assessment Analysis at Global, Regional, and Country Levels. 2016 [cited 11 Jul 2018].

7. IPC Technical Working Group. Integrated Food Security Analysis Report for Uganda. IPC, Uganda; 2017 p. 80.

8. Kosek MN, Ahmed T, Bhutta Z, Caulfield L, Guerrant R, Houpt E, et al. Causal Pathways from Enteropathogens to Environmental Enteropathy: Findings from the MAL-ED Birth Cohort Study. EBioMedicine. 2017;18: 109–117. doi:10.1016/j.ebiom.2017.02.024

9. Wegner CW, Loechl C, Mokhtar N. Foreword: Moderate acute malnutrition: Uncovering the known and unknown for more effective prevention and treatment. Food Nutr Bull. 2015;36: 3S-8S.

10. Hoppe C, Mølgaard C, Michaelsen KF. Cow’s milk and linear growth in industrialized and developing countries. Annu Rev Nutr. 2006;26: 131–173. doi:10.1146/annurev.nutr.26.010506.103757

11. Lee R, Singh L, van Liefde D, Callaghan-Gillespie M, Steiner-Asiedu M, Saalia K, et al. Milk Powder Added to a School Meal Increases Cognitive Test Scores in Ghanaian Children. J Nutr. 2018 [cited 5 Jul 2018]. doi:10.1093/jn/nxy083

12. Hoppe C, Mølgaard C, Dalum C, Vaag A, Michaelsen KF. Differential effects of casein versus whey on fasting plasma levels of insulin, IGF-1 and IGF-1/IGFBP-3: results from a randomized 7-day supplementation study in prepubertal boys. Eur J Clin Nutr. 2009;63: 1076–1083.

13. Grenov B, Briend A, Sangild PT, Thymann T, Rytter MH, Hother A-L, et al. Undernourished Children and Milk Lactose. Food Nutr Bull. 2016 [cited 23 Feb 2016]. doi:10.1177/0379572116629024

14. Hoppe C, Andersen GS, Jacobsen S, Mølgaard C, Friis H, Sangild PT, et al. The use of whey or skimmed milk powder in fortified blended foods for vulnerable groups. J Nutr. 2008;138: 145S-161S.

15. Maleta KM, Phuka J, Alho L, Cheung YB, Dewey KG, Ashorn U, et al. Provision of 10–40 g/d Lipid-Based Nutrient Supplements from 6 to 18 Months of Age Does Not Prevent Linear Growth Faltering in Malawi. J Nutr. 2015;145: 1909–1915. doi:10.3945/jn.114.208181

16. Stobaugh HC, Ryan KN, Kennedy JA, Grise JB, Crocker AH, Thakwalakwa C, et al. Including whey protein and whey permeate in ready-to-use supplementary food improves recovery rates in children with moderate acute malnutrition: a randomized, double-blind clinical trial. Am J Clin Nutr. 2016;103: 926–933. doi:10.3945/ajcn.115.124636

17. Fabiansen C, Yaméogo CW, Iuel-Brockdorf A-S, Cichon B, Rytter MJH, Kurpad A, et al. Effectiveness of food supplements in increasing fat-free tissue accretion in children with moderate acute malnutrition: A randomised 2 × 2 × 3 factorial trial in Burkina Faso. PLOS Med. 2017;14: e1002387. doi:10.1371/journal.pmed.1002387

18. Emanuelli T, Milbradt BG, Kolinski Callegaro M da G, Augusti PR, et al. Chapter 19 - Wheat Bran and Cadmium in Human Health. In: Watson RR, Preedy VR, Zibadi S, editors. Wheat and Rice in Disease Prevention and Health. San Diego: Academic Press; 2014. pp. 241–260. doi:10.1016/B978-0-12-401716-0.00019-2

19. Hess SY, Abbeddou S, Jimenez EY, Somé JW, Vosti SA, Ouédraogo ZP, et al. Small-Quantity Lipid-Based Nutrient Supplements, Regardless of Their Zinc Content, Increase Growth and Reduce the Prevalence of Stunting and Wasting in Young Burkinabe Children: A Cluster-Randomized Trial. PLOS ONE. 2015;10: e0122242. doi:10.1371/journal.pone.0122242

20. Cichon B, Fabiansen C, Iuel-Brockdorf A-S, Yaméogo CW, Ritz C, Christensen VB, et al. Impact of food supplements on hemoglobin, iron status, and inflammation in children with moderate acute malnutrition: a 2 × 2 × 3 factorial randomized trial in Burkina Faso. Am J Clin Nutr. 2018;107: 278–286. doi:10.1093/ajcn/nqx050

21. Kumordzie SM, Adu-Afarwuah S, Arimond M, Young RR, Adom T, Boatin R, et al. Maternal and Infant Lipid-Based Nutritional Supplementation Increases Height of Ghanaian Children at 4–6 Years Only if the Mother Was Not Overweight Before Conception. J Nutr. 2019;149: 847–855. doi:10.1093/jn/nxz005

22. Stewart CP, Dewey KG, Lin A, Pickering AJ, Byrd KA, Jannat K, et al. Effects of lipid-based nutrient supplements and infant and young child feeding counseling with or without improved water, sanitation, and hygiene (WASH) on anemia and micronutrient status: results from 2 cluster-randomized trials in Kenya and Bangladesh. Am J Clin Nutr. 2019;109: 148–164. doi:10.1093/ajcn/nqy239

23. Das JK, Salam RA, Hadi YB, Sadiq Sheikh S, Bhutta AZ, Weise Prinzo Z, et al. Preventive lipid-based nutrient supplements given with complementary foods to infants and young children 6 to 23 months of age for health, nutrition, and developmental outcomes. Cochrane Developmental, Psychosocial and Learning Problems Group, editor. Cochrane Database Syst Rev. 2019 [cited 14 Jun 2019]. doi:10.1002/14651858.CD012611.pub3

24. Style S, Tondeur M, Grijalva-Eternod C, Pringle J, Kassim I, Wilkinson C, et al. Assessment of the effectiveness of a small quantity lipid-based nutrient supplement on reducing anaemia and stunting in refugee populations in the Horn of Africa: Secondary data analysis. PLoS ONE. 2017;12. doi:10.1371/journal.pone.0177556

25. Batra P, Schlossman N, Balan I, Pruzensky W, Balan A, Brown C, et al. A Randomized Controlled Trial Offering Higher- Compared with Lower-Dairy Second Meals Daily in Preschools in Guinea-Bissau Demonstrates an Attendance-Dependent Increase in Weight Gain for Both Meal Types and an Increase in Mid-Upper Arm Circumference for the Higher-Dairy Meal. J Nutr. 2016;146: 124–132. doi:10.3945/jn.115.218917

26. WHO, FAO, editors. Vitamin and mineral requirements in human nutrition. 2nd ed. Geneva : Rome: World Health Organization ; Food and Agriculture Agency; 2004.

27. de Beer H. Dairy products and physical stature: a systematic review and meta-analysis of controlled trials. Econ Hum Biol. 2012;10: 299–309. doi:10.1016/j.ehb.2011.08.003

28. Fabiansen C, Phelan KPQ, Cichon B, Yaméogo CW, Iuel-Brockdorff A-S, Kurpad A, et al. Short Malnourished Children and Fat Accumulation With Food Supplementation. Pediatrics. 2018;142. doi:10.1542/peds.2018-0679

29. Sachdeva S, Dewan P, Shah D, Malhotra RK, Gupta P. Mid-upper arm circumference v. weight-for-height Z-score for predicting mortality in hospitalized children under 5 years of age. Public Health Nutr. 2016; 1–8. doi:10.1017/S1368980016000719

30. Subramanian SV, Ackerson LK, Smith GD. Parental BMI and Childhood Undernutrition in India: An Assessment of Intrauterine Influence. PEDIATRICS. 2010;126: e663–e671. doi:10.1542/peds.2010-0222

# Appendices

Appendix 1: Proposed list of study sites within the Busoga Subregion in Eastern Uganda

| District | Subcounty | Estimated number of parishes | Number of villages | Estimated households (2014) |
| --- | --- | --- | --- | --- |
| Mayuge | **13** | **56** | **512** | **97 392** |
|  | Wabulungu (HCIII) |  | | |
|  | Bukutube (HCII) |  |  |  |
|  | Malongo (HCIII) |  |  |  |
|  | Buwaiswa (HCIII) |  |  |  |
| Jinja | **11** | **69** | **659** | **105,358** |
|  | Busedde (HCIII) |  | | |
|  | Buwenge (HCIV)* |  |  |  |
|  | Walukuba (HCIV)* |  |  |  |
|  | Budondo (HCIV) |  |  |  |
| With the permission of the District health officers of each area, we have made this preliminary selection after visiting the sites. * The two sites from where recruitment will begin. | | | | |
