# Supplementary material for: Effect of milk protein and whey permeate in large quantity lipid-based nutrient supplement on linear growth and body composition among stunted children: A randomized 2 × 2 factorial trial in Uganda
Source: PLoS Med. 2023 May 23;20(5):e1004227. doi: 10.1371/journal.pmed.1004227 (PMC10204948; doi:10.1371/journal.pmed.1004227)
Supplement: S3 Text — (PDF) [file pmed.1004227.s008.pdf]

Statistical Analysis Plan

MAGNUS

The role of milk protein and whey permeate in the growth and development of stunted children: a randomised controlled trial in Eastern Uganda.

Trial ID: D222

ISRCTN: 13093195

Date: 15th April 2021

Signature____

Benedikte Grenov, Associate professor, Investigator on MAGNUS
Department of Nutrition, Exercise and Sports, UCPH

Signature____

Christian Ritz, Associate professor in statistics,
Department of Nutrition, Exercise and Sports, UCPH

Signature____

Henrik Friis, Professor, PI and Sponsor representative on MAGNUS,
Department of Nutrition, Exercise and Sports, UCPH

Introduction

This statistical analysis plan describes the statistical analyses to be carried out for the MAGNUS study described in the approved protocol dated 3rd December 2019 (V05).

This statistical analysis plan covers the primary and secondary outcomes described in the protocol, but not the exploratory outcomes.

Study design

The MAGNUS study (Milk affecting growth, cognition and the gut in child stunting), is a randomized, double-blind, two-by-two factorial trial, testing the effects of milk protein (MP) and whey permeate (WP) in large quantity lipid-based nutrition supplement (LNS-LQ). An un-supplemented group is included and used in secondary analyses. For a 12-week period, 750 stunted 1-5 year-old Ugandan children were randomized to one of four formulations of LNS-LQ as a daily supplement (n=4x150=600), or to continue with the family diet (n=150)(see Figure 1). All participants were followed-up at the same intervals throughout the intervention period (see Figure 2). This design allows assessment of the individual and combined effects of MP and WP among the 600 children allocated LNS, based on the factorial design: If the effects are independent, then we can compare the 300 given LNS with MP to the 300 given LNS without MP. And likewise, we can compare the 300 given LNS with WP to the 300 given LNS without WP. If the effects are not independent, then we will compare the four combinations pair-wise. In secondary analyses, we will be able to assess the effect of LNS by comparing the 600 given LNS, irrespective of milk ingredients, to the 150 given no supplements.

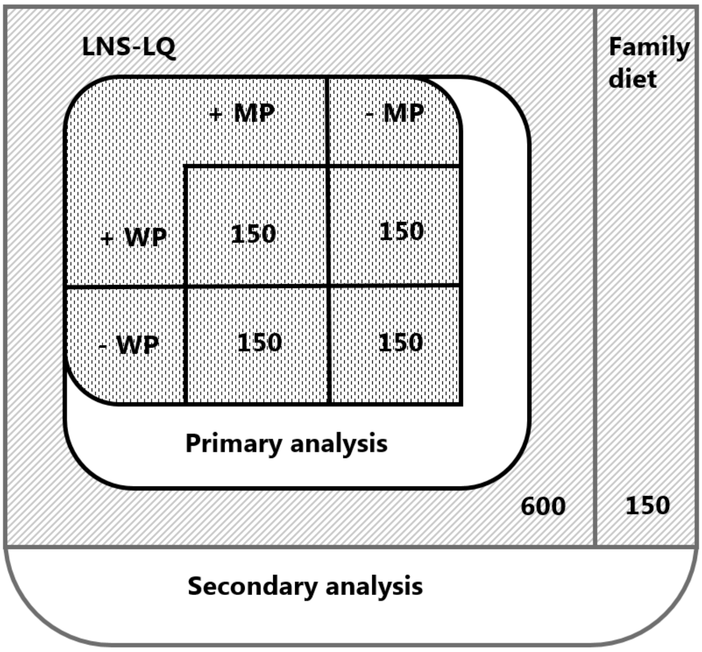

Figure 1. Primary analysis will compare large-quantity lipid-based nutrient supplements (LNS-LQ) with and without milk protein (MP) and whey permeate (WP) in a two-by-two factorial design with 150 participants in each given combination. Secondary analysis will compare all LNS-LQ interventions (n=600) to the reference group (family diet, n=150).

Definition of study population data sets

Intention-to-treat based on available cases: Randomized subjects, regardless of their intake of LNS or any protocol deviations.

Per-protocol: Randomized subjects with a minimum intake of the LNS-LQ of 80% during the 12-week intervention period and who have no other major protocol deviations with implications on the primary outcome.

General statistical considerations

All statistical analyses will be assessed using a 5% significance level and the statistical software R and Stata will be used.

Outcomes

All primary and selected secondary outcomes are measured at baseline, week 2, week 4, week 8 and week 12. Secondary outcomes of child development, bioimpedance, head circumference and haemoglobin are measured at baseline and at week 12 (see figure 2).

Primary outcome variables

There are two primary outcomes: Knee-heel length (mm) and total length (cm).

Secondary outcome variables

Mid-upper arm circumference (cm)

Height-for-age z-score (HAZ)

Weight-for-height z-score (WHZ)

Weight-for-age z-score (WAZ)

Gross motor, fine motor, language and socio-emotional development z-scores based on the Malawian Development Assessment Tool (MDAT)

Head circumference (cm)

Weight (g)

Haemoglobin (g/L)

Body composition

Fat mass (kg), fat-free mass (kg), fat mass index (kg/m2), fat-free mass index (kg/m2)

Skinfold thickness: triceps, subscapularis (mm)

Statistical analyses of MAGNUS primary and secondary outcomes

Primary outcomes

For the primary outcomes, the primary analyses will estimate the effects of the milk ingredients, based on the factorial trial, among the 600 given LNS-LQ. In secondary analyses, the effect of LNS-LQ, irrespective of milk ingredients will be estimated among all 750 children.

The primary analyses will be modified ITT analyses that evaluate efficacy based on available-case data. In addition, per protocol-analyses will be carried out.

Primary effect estimate: Difference in change in knee-heel length and total length between the LNS-LQ interventions from baseline to week 12.

Statistical analyses: Separate statistical analyses will be carried out for each of the two primary outcomes. For both outcomes, linear mixed models will be fitted such that the baseline value of the outcome will be included as part of the dependent variable. The analysis of covariance (ANCOVA) type of models will include three-way interactions between time and the two interventions: milk protein and whey permeate. The models will *a priori* be adjusted for the following covariates and potential confounders: age, sex and season as fixed effects, and participant ID and site as random effects to accommodate unsystematic differences between participants and sites, respectively. If other covariates or potential confounders show baseline imbalance, these will be included as fixed effects in the primary model.

The first step in the statistical analysis will be to test the null hypothesis of no interactions, implying that the above initial models may be reduced by replacing the three-way interaction term by the three main effects terms for time and the two interventions (milk protein and whey permeate). If the test of interaction is insignificant then estimated differences in changes over the 12 week intervention period with corresponding 95% confidence intervals will be reported for each of the two interactions. If the test of interaction is significant then pairwise comparisons between relevant combinations of the two interventions will be carried out using approximate t-tests and differences (of differences) in changes over the 12 week intervention period with corresponding 95% confidence intervals will be reported. Data-driven p-value adjustment for multiplicity will be applied to the pairwise comparisons, utilizing the correlations between test statistics (Hothorn et al., 2008). Additional data-driven multiplicity adjustment will be applied across the two primary outcomes, utilizing the correlation between the two primary outcomes (leading to a much less conservative adjustment than the Bonferroni adjustment).

For both primary outcomes, subgroup analyses based on baseline HAZ-score, breastfeeding, inflammation status, and sex will be carried out using the same type of linear mixed models, but replacing the three-way interaction term by a four-way interaction obtained by augmenting the three-way interaction through inclusion of the subgroups. Initially, tests for effect modification will be used to assess if significant differences between subgroups are present. The subsequent model reduction will proceed as detailed above for the primary analyses.

Handling of missing values: No imputations will be carried out.

Sensitivity analyses of the primary outcomes include: Unadjusted models, models adjusted for other sources of baseline imbalance than age, sex and season.

Secondary analysis: In addition, as shown in figure 1, the effect of the intervention vs. no intervention on the primary outcomes will be assessed using the same linear mixed models as described above.

Secondary outcomes

Modified ITT analyses will be evaluating efficacy based on available-case data. In addition, per protocol-analyses will be carried out.

Main effect estimate: Difference in change in HAZ, WHZ, WAZ, weight, mid-upper arm circumference, head circumference, body composition (fat mass, fat-free mass, fat mass index, fat-free mass index, triceps and subscapular skinfolds) between the LNS-LQ interventions from baseline to week 12. Difference in change in haemoglobin levels between LNS-LQ intervention (n=600) vs. the reference group (n=150) from baseline to week 12.

Secondary outcomes will in part be measured at fewer time points (see figure 2 below). However, the same ANCOVA-type linear mixed models as defined for the primary outcomes will still be applicable and model reduction and reporting will be done the same way as for the primary outcomes except that no adjustment for multiplicity will be used. Covariate adjustments and sensitivity analyses will be similar to the primary outcomes. Secondary analyses will assess the difference in change in HAZ, WHZ, WAZ, weight, mid-upper arm circumference, head circumference, body composition (fat mass, fat-free mass, fat mass index, fat-free mass index, triceps and subscapular skinfolds) between the LNS intervention (n=600) and the non-supplemented reference group (n=150) from baseline to week 12. In case of right-skewed data distributions, the outcome values will be logarithm-transformed prior to the statistical analysis.

References

1. Torsten Hothorn, Frank Bretz and Peter Westfall (2008). Simultaneous Inference in General Parametric Models. Biometrical Journal 50(3), 346--363

Plans for data collection

Activity			S	T_0	T_2	T_4	T_6	T_8	T_{10}	T_{12}
			**Time-point (weeks)**							
**Referral**		Screening for referral	▪							
**Enrolment**		Screening for eligibility		▪						
		Informed consent		▪						
		Nutrition counselling		▪						
		Allocation		▪						
**Data collection**	Primary outcomes	Knee-heel length		▪	▪	▪		▪		▪
		Height	▪	▪	▪	▪		▪		▪
	Secondary outcomes	Weight	▪	▪	▪	▪		▪		▪
		MUAC	▪	▪	▪	▪		▪		▪
		Head circumference		▪						▪
		MDAT assessment		▪						▪
		Bioimpedance		▪						▪
		Skinfolds (Triceps and subscapular)		▪	▪	▪		▪		▪
	Tertiary outcomes	Blood sample collection		▪						▪
		Stool sample collection		▪						▪
		Full clinical examination		▪						
		Clinical review			▪	▪		▪		▪
	Baseline characteristics	Demographics		▪						
		Household WASH assessment		▪						
		Dietary intake assessment		▪						
		Family care indicators		▪						
		Maternal anthropometrics		▪						
**Intervention**		LNS-LQ supplement provision		▪	▪	▪	▪	▪	▪	
		Reference group gift allocation		▪	▪	▪	▪	▪	▪	
**Adherence**		LNS-LQ empty sachet collection			▪	▪	▪	▪	▪	▪
		Phone follow-up appointment reminders		▪						▪
		Home visit		▪						▪

Figure 2: MAGNUS data collection time-points and visits. Timeline and visit overview for participants enrolled in the study. Phone follow-up and home visits are carried out as required and thus are unfixed time points. All participants are invited to the same follow-up visits. Time points are considered valid if taken within ±7 days of baseline, week 2 and week 4 and ±14 days from all other time points. MUAC: Mid-upper arm circumference; LNS-LQ: Large quantity lipid based nutrient supplement; S: Screening in village for referral; T: Week of visit from baseline (T0) to discharge (T12); WASH: Water, sanitation and hygiene Haemoglobin is a secondary outcome.
