# Supplementary material for: Effect of milk protein and whey permeate in large quantity lipid-based nutrient supplement on linear growth and body composition among stunted children: A randomized 2 × 2 factorial trial in Uganda
Source: PLoS Med. 2023 May 23;20(5):e1004227. doi: 10.1371/journal.pmed.1004227 (PMC10204948; doi:10.1371/journal.pmed.1004227)
Supplement: Attachment — Submitted filename: Response to reviewers_2023_02_21.docx [file pmed.1004227.s009.docx]

**Response to reviewers**

Manuscript "Effect of milk protein and whey permeate in large-quantity lipid-based nutrient supplement on linear growth and body composition among children with stunting: a randomised 2x2 factorial trial in Uganda" (PMEDICINE-D-22-03930R1)

Requests from the editors:

GENERAL

Please respond to all editor and reviewer comments detailed below, in full.

*Response: Thanks a lot for the very constructive and positive comments to our manuscript. We have done our best to address all the editor and reviewer comments, and think that it has improved the quality of our paper We believe all editor and reviewer comments have been addressed.*

Please complete the CONSORT checklist and ensure that all components of CONSORT are present in the manuscript, including [how randomization was performed, allocation concealment, blinding of intervention, definition of lost to follow-up, power statement].

When completing the checklist, please use section and paragraph numbers, rather than page numbers as these often change in the event of publication.

*Response: The CONSORT checklist has been completed according to the guideline and a definition of loss to follow-up has been included in the methods section, line 230-231.*

COMMENTS FROM THE ACADEMIC EDITOR

The trial is interesting and looks solid, notwithstanding industry funding, the authors have been fair and transparent in their findings One question is why this trial was done in older infants (mean age (30 months)?

*Response:*

*Thanks a lot. As for the comment about industry funding, please note that we did not get funding directly from the industry, but from Arla Food for Health, which is a public-private research partnership between the University of Copenhagen, Aarhus University and the dairy company, Arla. Yet, we made sure that “The funders of the study and the manufacturer of the LNS formulations had no role in study design, data collection, data analysis, data interpretation, or writing of the report”(PLOS Medicine submission form).*

*We wanted to assess if it is possible to obtain catch-up growth in stunted children beyond 2 years of age. To avoid interfering with breastfeeding, we selected a population from 12-59 months. We have included this explanation in the paragraph about study participants, line 162-170.*

Further, is 12 weeks a reasonable follow up given all the questions about sustained benefits?

*Response: We agree that 12 weeks is a rather short study duration; nevertheless, it was sufficient to show effects of LNS versus no LNS. In addition, we do not have any follow-up after the 12-week intervention stopped. Catch-up growth may take long and it is important to know the long-term effects of interventions. We have included the 12 weeks duration of the intervention and lack of long-term follow-up as a limitation in the discussion. Regarding post-intervention follow-up, even an additional 12 weeks would have been relatively short. Therefore, we are currently planning to do a 3 year follow-up study.*

ABSTRACT

Please report your abstract according to CONSORT for abstracts, following the PLOS Medicine abstract structure (Background, Methods and Findings, Conclusions, as below)

<https://eur02.safelinks.protection.outlook.com/?url=http%3A%2F%2Fwww.consort-statement.org%2Fextensions%3FContentWidgetId%3D562&data=05%7C01%7Cbgr%40nexs.ku.dk%7C2ad2ea77ab47407872d808db039361e5%7Ca3927f91cda14696af898c9f1ceffa91%7C0%7C0%7C638107703766122989%7CUnknown%7CTWFpbGZsb3d8eyJWIjoiMC4wLjAwMDAiLCJQIjoiV2luMzIiLCJBTiI6Ik1haWwiLCJXVCI6Mn0%3D%7C3000%7C%7C%7C&sdata=fKRsDeq4%2BAsiOLzehQPYfzbEAJVHuHRs8pn75dRz6s4%3D&reserved=0>

*Response: Thanks, the abstract has been revised to follow the CONSORT guideline and PLOS Medicine abstract structure. Methods and results in the previous version of the manuscript have been combined under the heading Methods and Findings. The background and description of the study location have been expanded, the blinding of participants, investigators and outcome assessors has been included and adjustments of statistical analyses have been added. Distribution of drop-outs between study groups has been included in the results as well as a more detailed description of adverse events and main limitations of the trial. The conclusion has been updated to be more specific as suggested by the editor.*

Please structure your abstract using the PLOS Medicine headings (Background, Methods and Findings, Conclusions).

Please combine the Methods and Findings sections into one section, “Methods and findings”.

*Response: Methods and Findings have been combined into one paragraph.*

Abstract Background: It would be helpful to elaborate on some of the context here. What do we know about the different supplements, why these specifically? Why is the comparison of these three supplements an important one to make? For example

*Response: We have expanded the abstract background to emphasize that milk protein and whey permeate are different milk components. Studies have generally assessed the effect of milk or whey protein or skimmed milk powder (milk protein + lactose) on growth and body composition, but not the separate effects of milk protein and lactose. We hope this is sufficient to clarify the background for the reader in the limited space available in an abstract. However, we give more information in the main text.*

Abstract Methods and Findings:

Line 7: Please revise the sub-heading to “Methods and Findings” (as above). Suggest “We performed a randomized…” or something similar Please include further details of population/setting rural Vs urban for example.

Any further details on the community-based nature of the study? Were community clinics involved? What kind? For example – this was done rather well in the methods section of the main manuscript

*Response: We have rephrased the start of the paragraph. Unfortunately we had to leave out a more detailed description of the population/setting to keep the abstract below 500 words. It now reads (Line 7-9): “We performed a randomized, double-blind, 2x2 factorial trial among 12-59 months-old stunted children in Uganda”.*

Please specify who was blinded to the intervention and control.

*Response: Thanks, this has been specified (line 11-12)*: *Investigators and outcome assessors were blinded, however participants were only blinded to the ingredients in LNS.*

Please provide the number of participants lost to follow up in each group.

*Response: We agree that numbers analysed in each group should be reported according to CONSORT Abstract. However, as the number of participants lost to follow-up is very low in this trial and we use all available data at each time point for the linear mixed regression models, we think it may be complex to specify all these numbers. We suggest to rephrase the sentence to specify that loss to follow-up was evenly distributed between groups: 736 (98.1%), evenly distributed between study groups, completed the 12-week follow-up*

Please define all abbreviations at first use including those for statistical reporting IQR, SD, CI, for example and those used for weights and heights.

*Response: Thanks, all abbreviations have now been defined at the first occurrence.*

Please quantify the main results with p values, as well as 95% CIs.

*Response: P-values have been added to all the main results, except for the proportion of weight gain that is due to fat-free mass, as a p-value is not applicable.*

When reporting p values please report as p<0.001 (not .001) or where higher as p=0.002 (not .002), for example Suggest revising statistical reporting, including throughout the main manuscript, to read as follows:

“0.06 (95%CI [0.02,0.10]; p<0.001)” note the addition of parentheses around CIs and the altered use of semi-colons/colons Please ensure that all numbers presented in the abstract are present and identical to numbers presented in the main manuscript text.

*Response: Results have been updated to follow the suggested reporting structure both in the abstract and in the results section.*

Please include any variable factors that were adjusted for in your analyses.

*Response: Adjustments have been added to the description of statistical analyses: Data were analysed based on intention-to-treat using linear mixed-effects models adjusted for age, sex, season and site.*

Please include a summary of adverse events that you refer to.

*Response: The adverse event description has been expanded to include a summary of the type of adverse events collected in the trial (line 22-24): Eleven serious adverse events occurred in 10 (1.3%) children, mainly hospitalization with malaria and anaemia, all deemed unrelated to the intervention.*

In the last sentence of the Abstract Methods and Findings section, please describe the main limitation(s) of the study's methodology.

*Response: A sentence with methodological limitations has been included (line 42-43): Main limitations were lack of blinding of caregivers and short study duration.*

Line 23-25 – suggest revising this paragraph for improved clarity, especially for the general reader.

*Response: Thanks, we have now expanded the paragraph to explain to the general reader that lack of intervention between MP and WP allowed us to analyze the main effects of these ingredients separately.*

Line 146: “…above and below 2 years, children aged 12-59 months…” but here (in the abstract) you refer to the median/IQR age range only. Suggest additional details here for clarity to prevent confusion perhaps?

*Response: Thank you for this comment. We agree this could be misunderstood. We have included children from 12-59 months without aiming at a specific distribution of numbers below and above 2 years and without pre-specifying subgroup analyses for children below and above 2 years. The manuscript has been updated to reflect this.*

Abstract Conclusions:

Please interpret the study based on the results presented in the abstract, emphasizing what is new without overstating your conclusions.

* Please avoid vague statements such as "these results have major implications for policy/clinical care". Mention only specific implications substantiated by the results

*Response: The conclusion has been updated to be more specific (line 44-50): “Adding dairy to LNS has no additional effects on linear growth or body composition in stunted children aged 12-59 months. However, supplementation with LNS, irrespective of milk, supports linear catch-up growth and accretion of fat-free mass, but not fat mass. If left untreated, children already on a stunting trajectory gain fat at the expense of fat-free mass, thus nutrition programs to treat such children should be considered.”*

AUTHOR SUMMARY

Thank you for including an author summary which requires revision. The author summary should follow a similar structure as the abstract (and main manuscript) introducing the need for the study, the details of the study and its findings and finally the study outcomes and how these may impact the bigger picture.

Please see below for further guidance.

<https://eur02.safelinks.protection.outlook.com/?url=https%3A%2F%2Fjournals.plos.org%2Fplosmedicine%2Fs%2Frevising-your-manuscript%23loc-author-summary&data=05%7C01%7Cbgr%40nexs.ku.dk%7C2ad2ea77ab47407872d808db039361e5%7Ca3927f91cda14696af898c9f1ceffa91%7C0%7C0%7C638107703766122989%7CUnknown%7CTWFpbGZsb3d8eyJWIjoiMC4wLjAwMDAiLCJQIjoiV2luMzIiLCJBTiI6Ik1haWwiLCJXVCI6Mn0%3D%7C3000%7C%7C%7C&sdata=sbjsCGt1OR%2FW7FN5dIQhUvuUUhYNUd2aAXhUKMA4Xks%3D&reserved=0>

It may also help you to review some examples from recently published manuscripts which you can find on our homepage here: <https://eur02.safelinks.protection.outlook.com/?url=https%3A%2F%2Fjournals.plos.org%2Fplosmedicine%2F&data=05%7C01%7Cbgr%40nexs.ku.dk%7C2ad2ea77ab47407872d808db039361e5%7Ca3927f91cda14696af898c9f1ceffa91%7C0%7C0%7C638107703766122989%7CUnknown%7CTWFpbGZsb3d8eyJWIjoiMC4wLjAwMDAiLCJQIjoiV2luMzIiLCJBTiI6Ik1haWwiLCJXVCI6Mn0%3D%7C3000%7C%7C%7C&sdata=ftjw3ghy56rWB3iRD7sZAHeWzXHYjHqdHgukbhlVqDM%3D&reserved=0>

*Response: Thank you. The author summary - especially “Why was this study done?” - has been revised to match PloS Medicine guidelines. It now reads (line 68-78):*

- *Stunting affects 149 million children below 5 years and is associated with increased morbidity and mortality, delayed cognitive development and later risk of chronic diseases*
- *There are concerns that catch-up growth beyond 2 years may not be possible and that supplementation of already stunted children may lead to fat mass accretion*
- *Nutritional interventions using small or medium quantity lipid-based nutrient supplement (LNS) to prevent stunting among children below 2 years have had only small or modest effects and no trials have evaluated the effect of LNS among children already stunted.*
- *Milk may support growth and fat-free mass accretion. However, it is expensive and individual effects of milk protein and whey permeate are unknown*
- *We aimed to assess the effects of milk protein and whey permeate in large quantity LNS among 1-5 year-old children that were already stunted*

METHODS and RESULTS

Please move the ethics statement to the end of the methods section of the main manuscript

*Response: Thanks, the ethics paragraph has been moved to the end of the methods section*

There are a lot of abbreviations used throughout the manuscript, please consider re-defining some of these at first use in the methods section for example where you describe the study design at line 136 onwards.

Please also ensure that all abbreviations introduced later in the manuscripts are also clearly defined – ID (line 168) and KHz (line 231), SD (line 251) for example. Please check carefully throughout and amend where necessary

*Response: We regret the oversight and have updated the abbreviation definitions and list.*

Please include the study protocol document and analysis plan, with any amendments, as Supporting Information to be published with the manuscript if accepted.

*Response: The study protocol and statistical analysis plan have been uploaded with the revised manuscript.*

Please specific in the main methods section that analysis is intention to treat (as for the abstract) Line 251: “To detect a 0.35 SD…” please define SD for the reader, please also see comments below regarding tables.

*Response: Intention-to-treat analysis is specified in line 296 and SD is defined in line 105.*

TABLES

Please ensure all tables are associated with an appropriate caption/legend which clearly describes their content without the need to refer to the manuscript text. Please ensure that any and all abbreviations including those used for statistical reporting are defined within the caption/legend

Throughout, please replace semicolons, used to differentiate upper and lower bounds, with commas

*Response: Semicolons have been replaces with commas in all 95% confidence intervals and interquartile ranges.*

Table 2: baseline characteristic reporting utilises a variety of different units but the footnote only helps partially because it does not clearly detail what applies where – please revise accordingly – I note superscript text delineating the units used rather inconsistently placed and not clearly defined in the footnote.

*Response: Thank you for this comment. It was an omission on our side. The footnote has now been updated.*

Tables 3 and 4 – please indicate whether your analyses are adjusted or unadjusted and in the event of adjustments please state in the captions what factors are adjusted for. In addition, for the purpose of transparent data reporting please also include the unadjusted analyses for comparison.

*Response: Table 3 includes unadjusted values from paired t-tests. Since we are comparing the same children between two time points, adjustments are not meaningful. This has now been specified in the table footnote. Table 4 includes pre-specified adjustments. Unadjusted values have been added in Supplementary table 1 (S1 Table).*

SUPPLEMENTARY TABLES

Please ensure that each has an appropriate caption which clearly details the table contents without the need to refer to the text. Please revise the presentation of data throughout. It is not clear to the reader what the numerical values within the column between p values and 95% CIs represent

*Response: Supplementary tables have been simplified to make them clearer to the reader. In supplementary table 1 and 5, data are presented as estimate B (95% CI), p-value. For the supplementary tables with subgroup analysis, we have removed the B (95%CI) for the difference between strata, so that we now only show the p for interaction and then the stratum-specific B (95%CI).*

Please replace semicolons with commas when reporting upper and lower bounds

*Response: Semicolons have been replaced with commas*

In places there are closing parentheses in the absence of opening parentheses, please include both. Suggest combining these columns for each sub-group, re-labelling the column header and reporting as follows – for example, row 1, columns 2 and 3 should be combined to a single column and read: -0.21 (-0.47, 0.05) Please check and revise throughout all the tables

*Response: Supplementary tables have been updated to accommodate the suggested changes as indicated above and the revised supplementary table 1-5.* Throughout, please indicate whether analyses are adjusted or unadjusted and detail which factors are adjusted for in the caption/legends. As above, where adjusted analyses are reported please also include unadjusted analyses for comparison.

*Response: Thanks, we have included additional supplementary tables with unadjusted values. We have also specified adjustments in the table footnotes. Please note that in the unadjusted analysis, we removed age, sex and season as covariates. However, for obvious technical reasons we had to keep baseline value of the outcome and the ID.*

FIGURES

Please ensure all figures are associated with an appropriate caption/legend which clearly describes their content without the need to refer to the manuscript text. Please ensure that any and all abbreviations including those used for statistical reporting are defined within the caption/legend

Figure 1- suggest revising the title to include details of what the flow chart shows.

*Response: The title of figure 1 has been changed to: “Trial profile showing the number of children assessed for stunting, screened, recruited to each intervention group and lost to follow-up. Loss to follow-up was defined as those not returning for the 12-week visit”. Please note that the number of children referred for eligibility assessment (n=1112) is correct. However, in the previous version of the manuscript, we mentioned that it was 1193 in the text. This has now been corrected (line 329).*

DISCUSSION

Please remove all sub-headings from the discussion such that the discussion reads as a single piece of continuous prose and please structure as follows: a short, clear summary of the article's findings; what the study adds to existing research and where and why the results may differ from previous research; strengths and limitations of the study; implications and next steps for research, clinical practice, and/or public policy; one-paragraph conclusion.

*Response: Subheadings have been removed, and the discussion and study limitations have been expanded according to comments from the editor and reviewer 1-3.*

Please remove funding/competing interests/data statements from the end of the discussion and include only in the manuscript submission form in the relevant sections. In the event of publication these will be compiled as metadata.

*Response: Thanks, these paragraphs have been removed from the main manuscript.*

REFERENCES

Please ensure that citations are placed within square parentheses which precede punctuation For example, line 75 should read: “Globally, 149 million children under 5 years are stunted [1].” Please check and revise throughout

*Response: Thanks, punctuations and commas have been moved to precede square parentheses.*

##################################################################################

Reviewer #1: This randomised, double-blind, community-based 2x2 factorial trial aims to assess the effects of MP and WP in lipid-based nutrient supplement (LNS), and of LNS itself, on linear growth and body composition among children with stunting.

Comments:

Can the authors please provide copies of the study protocol, pre-specified statistical analysis plan, and associated guideline checklist for this study?

*Response: The study protocol and signed statistical analysis plan can be found at (ISRCTN13093195) and has now been uploaded to the PloS Medicine submission portal.*

"Children were individually randomised to one of the four LNS formulations (1:1:1:1) or the reference group (4:1)."

and

"All investigators and outcome assessors were blinded with respect to LNS vs no LNS and to the ingredients contained in the differently coded LNS sachets. Caregivers were blinded with respect to the type of LNS allocated since the four formulations were indistinguishable in terms of appearance, smell and taste. "

The authors have followed a rigorous study design, helping to minimise potential sources of bias.

*Response: Thanks.*

"To detect a 0.35 SD or greater difference between any two groups at 5% significance level and 80% power while allowing for 10% attrition, we recruited 150 children per group (600 in total based on the 4 combinations of MP and WP). If there were no interactions between the two experimental arms, then two groups of 300 children could be compared enabling differences of 0.24 SD to be detected."

The authors have suitably provided the basis for the sample size calculation, with associated assessment of study power.

*Response: Thanks*

"Descriptive statistics were presented as mean (SD), median (interquartile range) for continuous variables and frequency % (n) for categorical variables. Mean changes in anthropometry and body composition among unsupplemented children were given with 95% confidence intervals and p value based on paired t-tests. The statistical analyses were based on the intention-to-treat (ITT) principle using available case data. The 2x2 factorial design was analysed using a linear mixed model, where we first tested for any interactions between the two interventions in the matrix (MP vs WP). In case of no interaction, we then tested for main effects on linear growth and body composition (i.e. +MP vs -MP and +WP vs -WP). In our secondary analysis, we also compared the intervention i.e. LNS irrespective of ingredient (600 participants) against the unsupplemented reference group (150 participants). We carried out prespecified subgroup analyses for breastfeeding status, sex, stunting severity, and inflammation (defined as serum AGP>1 g/l) to test for any further interactions."

The authors have applied technically appropriate statistical methods within the context of this research.

*Response: Thanks.*

Can the authors please clarify if and how they assessed distributional assumptions (e.g. normality and linearity) in the choice of application of statistical methods?

*Response: The statistical methods have been amended to clarify this (line 290-292): Descriptive statistics were presented as mean (SD) for normally distributed variables, median (interquartile range) for non-normally distributed continuous variables and frequency % (n) for categorical variables. Distribution was assessed visually using normal probability plots (line 311-312): For all analyses, model assumptions were assessed visually using residual and QQ plots.*

"All our analyses were adjusted for the baseline measure of the outcome, age, sex, and season as fixed effects, and site plus participant as random effects to obtain the adjusted measures at 95% confidence interval and p<0.05 significance level."

The authors have suitably adjusted the analyses for covariates, including the stratification variable site.

*Response: Thanks.*

"A per protocol analysis was then carried out including only those children who fulfilled the eligibility criteria and had minimum 80% compliance (supplemented group). Difference in compliance between LNS formulations, sex, age category, and breastfeeding status were assessed by chi-square tests."

The authors have also satisfactorily undertaken additional sensitivity and exploratory analyses, helping to demonstrate the robustness of the study findings.

*Response: Thanks.*

"Of 1193 stunted children referred to the study sites, 750 (63%) were enrolled and randomised to the five groups of which 736 (98.1%) completed the 12-week follow-up."

Can the authors please comment on whether the included cohort can be considered to be representative, for generalisability of the study findings?

*Response: It seems we made a mistake in the text the reviewer quoted. As correctly written in figure 1, the number referred for eligibility assessment was 1112. We have now corrected the sentence to: “Of 1112 stunted children referred to the study sites, 750 (67%) were enrolled ….”.*

*Of the 362 not included, 81 showed up, but were eligible. So, it is only 281/1112 (25%) that were not seen. Unfortunately, we don’t have any data on these children, so we are not able to say whether they were different from those assessed. It is possible that they come from families that were less well off, and that the children had worse nutritional status. If so, then the effects of the interventions would be greater than among those who were included. We think the potential for selection bias is quite low, and, if anything, we may have underestimated the true effect of the intervention. However, it is important to notice that we probably reduced the potential for selection bias by screening in communities, rather than at health facilities.*

"The randomisation resulted in baseline equivalence with respect to both the primary and secondary comparisons (Table 2)."

Can the authors please check if 'currently breastfed', 'diverse diet', and 'Malaria (RDT positive)' are sufficently balanced between groups, in particular between LNS and no supplement?

*Response: We agree there were some differences between groups for these baseline characteristics. We have included this in the updated manuscript: The randomisation resulted in baseline equivalence, except for currently breastfed, diverse diet and positive malaria test (****Table 2****).*

"models adjusted for baseline value, age, sex, season, site and ID2"

Whilst it is noted that the authors have conducted a subgroup analysis by breastfeeding, did the authors consider adjusting for the above named variables in the models as a sensitivity analysis?

*Response: We have performed sensitivity analyses as suggested and found that breastfeeding status, dietary diversity or a positive malaria test did not influence any of the primary or secondary study results. This has been added to the results section, line 365-367 and 406-408. The additional adjustments did not change the estimates.*

"Limitations include incomplete blinding of caregivers in regards to LNS supplementation."

Can the authors please expand on the discussion of the main study limitations?

*Response: We have expanded the text describing study limitations, see line 494-501.*

Overall, this is a well written article, with accurately presented Results.

*Response: Thanks.*

################################################################################

Reviewer #2: Thank you for this opportunity to review this well-written manuscript and preview the results from this trial. Some comments and thoughts below for your consideration:

1. The tables can be improved and be made stand alone by adding some footnotes such as definitions for terms like severe food insecurity and diet diversity in the main text. Same for supplementary tables. Supplementary Table 4 is missing a column of p-value for interaction between MP and WP.

*Response: Thank you. In table 2, severe food insecurity and diet diversity have been defined in the footnote. In addition, the methods section now specifies which tools we used. The aim of the Per Protocol analysis was merely to assess if the effects of MP and WP was different without individuals not adhering to the intervention. Hence it would not be meaningful to test for interactions. First, if there were interactions in the ITT but not PP, or vice versa, then we would still need to make the same models/comparison to see the effect of excluding non-adherent participants. Second, the ITT was pre-specified as our primary analysis, and if there were interactions in the PP analysis then it could have been due to selection bias/confounding.*

2. This may be a minor point - classically, a 2X2 trial assigns the first intervention and then randomizes within the two groups to the second intervention to result in four groups. The statistical analysis are appropriate but the methods indicate that the children were assigned to 5 separate arms and it will be good to be consistent or describe how the randomization sequence was operationalized to address this.

*Response: We made two randomization lists – one for each site that included all 5 intervention groups (10 codes, 2 for each group). We did not first assign one intervention and then randomize to the second intervention. What the reviewer suggest may work for other interventions (say, with two separate micronutrients and placebo). But we cannot see how we could have used that approach in this study, where we needed to include the two factors in the same LNS-product, and also had a fifth, unsupplemented group.*

3. I am also not sure re: the double blinded claim here since the control group and the outcome assessors knew they were not receiving any supplement? Perhaps elaborate in limitations - line #431.

*Response: Thanks for this comment. We have now specified how distribution of LNS and laundry soap was done to minimize risk of unblinding outcome assessors, see line 221-223: “Distribution was done at the end of the visit by the site pharmacist in a separate location. Caregivers carried LNS or laundry soap in a bag to minimize risk of unblinding investigators or outcome assessors”. In addition we have added the risk of unblinding outcome assessors to limitations, see 497-500.*

4. * Lines #251 and #255 - Please mention the significance or rational behind using 0.35 SD and 0.24 SD (or provide appropriate reference)

*Response: While there is no clear rationale for exactly 0.35 SD, such a difference (Cohen’s d) is considered to be a small-to-medium effect size of importance for public health that corresponded with a reasonable (and round) group size of 150. The other effect sizes of 0.24 and 0.27 is then a consequence of the other comparisons we found relevant, ie between 300 vs 300, and between 600 vs 150.*

* Line #264 For continuous variables, were the data distributed normally?

*Response: Thank you. We have specified how normal distribution was assessed and when mean (SD) and median (IQR) were used for descriptive presentation of data. Please see the statistical methods in the revised manuscript, line 290-292: Descriptive statistics were presented as mean (SD) for normally distributed variables, median (interquartile range) for non-normally distributed continuous variables and frequency % (n) for categorical variables. Distribution was assessed visually using normal probability plots..*

5. Line #295 - Data obtained according to age division (below/above 2y) not present in any of the tables.

*Response: Thank you for this comment. The manuscript was not clear about the inclusion of children above and below 2 years. We included children from 12-59 months without aiming at a specific distribution of numbers below and above 2 years and without pre-specifying subgroup analyses for children below and above 2 years. The manuscript has been updated to reflect this, see participant description, line 162-169 “The study was conducted from two study sites at Walukuba and Buwenge health centres, Jinja, East Central Uganda. With support from local village health teams, 12-59 month-old children with a HAZ <-2 and weight-for-height z-score (WHZ) ≥-3 were identified in villages surrounding the two health centres and referred to a study site for full assessment of eligibility. The age range was chosen to minimize interference with breastfeeding and to assess catch-up growth in children beyond 2 years of age”.*

################################################################################

Reviewer #3: I have read with great interest the manuscript by Mbabazi and colleagues on the effects of milk and whey protein in large quantity LNS supplements on height and body composition. The research group behind the research is well known for very well designed and executed studies, and this study is no exception.

*Thank you.*

The authors report that neither the addition of milk protein (MP) or whey protein (WP) had an additional effect on height gains in stunted children in Uganda. This finding is important, as milk is an expensive ingredient in ready-to-use supplements, and leaving out milk could potentially reduce cost, thereby enabling more children to be treated for the same budget.

*Response: We agree that cost is a high priority when it comes to development of new formulations to treat undernourished children. However, the main purpose of the MAGNUS trial was not to reduce cost, but to assess the separate effects of milk protein and lactose/minerals (whey permeate) on growth and body composition. We used expensive ingredients (e.g. milk protein isolate) that may not be compatible with large scale production and therefore we don’t think it will be right to discuss costs in the current manuscript.*

The manuscript is well-written and the results and conclusions are well presented. My main concern with the manuscript as it stands now is some discussion on the sample size. The authors were in a sense lucky in that there were no statistical interactions between MP and WP, enabling them to combine the groups, giving them approximately 300 children per group (MP vs no MP ; WP vs no WP). Looking at supplementary table 4, what strikes me is that for the MP vs no MP comparison, all anthropometric indicators are positive, while for the WP vs no WP comparisons, all of them (except FFMI) are negative. Even though none of them are statistically significant, the question arises whether this would also have been the case if the sample size would have been twice as large. The main conclusion of the paper, no impact of MP on height, would probably not have changed, but outcomes such as FFMI or WHZ might have become significant, as well as perhaps a negative effect of WP on HAZ. Hence, a paragraph in the discussion section on the sample size would be welcome.

*Response: Thank you, we have also noticed this. We have now mentioned the observation in the discussion, line 435-439: “Although there were no main effects of milk protein or whey permeate in the current study, it is noticeable that all effect sizes were positive for milk protein and negative for whey permeate. If this could be confirmed in other trials, LNS with milk ingredients containing milk protein but not whey permeate or lactose (e.g. skimmed milk powder) would be recommended.”*

Minor concerns

Review plos med

Abstract.

Lines 23-26. None of the effects of milk protein or whey protein on height or knee-heel length are statistically significant. I'm fine with the way these effects are presented. But I don't understand why then the effects of MP on FFMI need to be specified as 'non-significant'. Perhaps start this paragraph with stating that none of the effects of MP or WP were statistically significant. And then the specific mentioning of the effect of MP on FFMI can be omitted.

*Response: The non-significant effect of MP on FFMI has been deleted from the abstract.*

Line s31-32. A 0.21 kg increase in weight corresponding to an increase in how many Z-score (WAZ). And how many WHZ/WLZ scores?

*Response: The 0.21 kg increase in weight corresponded to 0.15 WAZ and 0.08 WHZ (table 4). As the abstract is already very long, we are unsure if we can add this information. However, we have included the corresponding increase in WHZ in the results section. We have not added the increase in WAZ as in our view both increase in height and weight will contribute to increase in WAZ and therefore this will not add much to the interpretation of the weight increase.*

Line 116 low income (2 words)

*Response: We have changed to low income throughout the manuscript.*

Line 121-122. While this hypothesis is valid in itself, most of the SQ-LNS trials reported in the paragraph above had cow milk as main protein source.

*Response: Yes we agree that many of the SQ-LNS supplements had milk as a major protein source. However, due to the small quantity (10-20 g/day), the amount of milk protein per day was considerably lower than the 7 g/day in large quantity LNS with 50% protein from milk. Increasing the amount of milk protein was one of our main hypotheses to increase the effect of LNS on stunting.*

Line 126 increases

*Response: We are uncertain about this comment. There is no mention of increase/increases in line 126. We have checked the use “increase” or “increases” in the Introduction and Methods and don’t seem to find any errors?*

Line 193. I'm fine with the randomisation of the 4 different LNS types, but how can you blind the study team to the 'no-LNS' group, as they will go home without LNS sachets

*Response: Thanks for this comment. We have now specified how distribution of LNS and laundry soap was done to minimize risk of unblinding outcome assessors, see line 221-223: “Distribution was done at the end of the visit by the site pharmacist in a separate location. Caregivers carried LNS or laundry soap in a bag to minimize risk of unblinding investigators or outcome assessors”. In addition we have added the risk of unblinding outcome assessors to limitations.*

Line 276 secondary analyses or In a secondary analysis

*Response: Thank you. In secondary analysis has been corrected to “in secondary analyses”.*

Line 294. Any justification for using AGP only to indicate systemic inflammation?

*Response: We used serum AGP to define inflammation since, in contrast to serum CRP, it is a slow-reacting acute phase reactant. This has now been specified in the manuscript. Please note that the cut-off was 1.2, not 1.0 as we wrote in the previous version.*
