# Supplementary material for: Effect of milk protein and whey permeate in large quantity lipid-based nutrient supplement on linear growth and body composition among stunted children: A randomized 2 × 2 factorial trial in Uganda
Source: PLoS Med. 2023 May 23;20(5):e1004227. doi: 10.1371/journal.pmed.1004227 (PMC10204948; doi:10.1371/journal.pmed.1004227)
Supplement: Attachment — Submitted filename: Response to reviewers - 2.docx [file pmed.1004227.s010.docx]

**Response to reviewers - 2**

Manuscript "Effect of milk protein and whey permeate in large-quantity lipid-based nutrient supplement on linear growth and body composition among children with stunting: a randomised 2x2 factorial trial in Uganda" (PMEDICINE-D-22-03930R1)

Requests from the editors:

GENERAL

Thank you for your very detailed and considered responses to previous editor and reviewer comments. Please see below for further comments from the editorial team.

*Response: Thank you.*

INDUSTRY FUNDING

Thank you for your clear response to this question. Please include the following “Arla Food for Health, is a public-private research partnership between the University of Copenhagen, Aarhus University and the dairy company, Arla.” Or similar, in the relevant section of the manuscript submission form.

*Response: The suggested sentence has been included in the Financial Disclosure. The paragraph now reads: “The trial was funded by Arla Food for Health, https://arlafoodforhealth.com/ (HF). Arla Food for Health, is a public-private research partnership between the University of Copenhagen, Aarhus University and the dairy company, Arla. Additional funds were obtained from the Danish Dairy Research Foundation, https://danishdairyboard.dk/research/ddrf/ (HF), Augustinus Fonden, https://augustinusfonden.dk/ (HF), Læge Sofus Carl Emil Friis og Hustru Olga Doris Friis’ Legat, no homepage available (HF), and A. P. Møller Fonden til Lægevidenskabens Fremme, https://www.apmollerfonde.dk/ansoegning/fonden-til-laegevidenskabens-fremme/ (HF). The funders of the study and the manufacturer of the LNS formulations had no role in study design, data collection and analysis, decision to publish, or preparation of the manuscript.”*

AUTHOR SUMMARY

Thank you for modifying the summary which reads very nicely but is a little too long – suggest the following revisions “Why was this study done”:

• Stunting affects 149 million children below 5 years and is associated with increased morbidity and mortality, delayed cognitive development and later risk of chronic diseases.

• There are concerns that catch-up growth beyond 2 years may not be possible and that supplementation of already stunted children may lead to fat mass accretion. But no trials have evaluated the effect of lipid-based nutrient supplements (LNS) among children already stunted.

• Milk may support growth and fat-free mass accretion. However, it is expensive and individual effects of milk protein and whey permeate are unknown.

• We aimed to assess the effects of milk protein and whey permeate in large quantity LNS among 1-5 year-old children that were already stunted.

“What did the researchers do and find”

• Line 68 – suggest a separate bullet point for sentence beginning “In contrast…”

*Response: The suggested changes have been included in the ATHOR SUMMARY.*

SOCIAL MEDIA

To help us extend the reach of your research, please provide any Twitter handle(s) that would be appropriate to tag, including your own, your co-authors’, your institution, funder, or lab.

Please detail any handles you wish to be included when we tweet this paper, in the manuscript submission form when you re-submit the manuscript.

*Response: We suggest using two twitter accounts from University of Copenhagen and Makerere University, respectively:*

*@NEXSKU*

*@Mak_Pediatrics*

*These two twitter accounts have been added to the submission form under social media*

Comments from Reviewers:

Reviewer #1: Many thanks to the authors for satisfactorily considering and responding to each comment in turn, undertaking additional sensitivity analyses and amending the reporting of the study accordingly.

*Response: Thank you.*

Reviewer #2: The responses appear to adequately address the concerns. However, the manuscript file sent for review was the previous version without the changes. Thanks.

*Response: Thank you.*
